# The feasibility of self-performing measurements of peripheral oxygen saturation and respiratory exercises in home-isolated COVID-19 patients—a single-arm prospective trial

**DOI:** 10.1186/s40814-023-01415-x

**Published:** 2023-12-02

**Authors:** Hans Joakim Myklebust-Hansen, Eivind Hasvik, Volker M. Solyga, Waleed Ghanima

**Affiliations:** 1 Department of Acute Medicine, Østfold Hospital Trust, 1714 Grålum, Norway; 2 Department of Physical Medicine and Rehabilitation, Østfold Hospital Trust, Grålum, Norway; 3 Department of Hematology, Oslo University Hospital and Institute of Clinical Medicine, University of Oslo, Oslo, Norway

**Keywords:** COVID-19, Self-measurements, Silent hypoxia, Respiratory exercises, Feasibility

## Abstract

**Background:**

COVID-19 is a highly contagious disease where isolation of infected individuals is deemed warranted. If possible, home isolation is preferred over hospitalization. This implies a need for methods of observation that can ensure the safety of these patients. Preventive treatment methods that can both decrease the probability for development of critical disease and hopefully decrease the need for hospitalization would be an added benefit. This was a single-arm prospective pilot study performed to assess the feasibility of performing self-measurements of SpO2 and respiratory exercises in at-home isolated COVID-19 patients.

**Method:**

A total of 40 ambulant SARS-CoV-2-positive individuals in home isolation were followed up for a period of 14 days. At baseline, they were equipped with a pulse oximeter, PEF meter, a project diary to note all measurements, and simple instructions on how to perform respiratory exercises. No other contact was made, but participants were instructed to contact the hospital based on given criteria for blood oxygenation levels and dyspnea severity and to return study equipment and the project diary at the end of study.

**Results:**

During the follow-up period, 35 participants (87.5%) recorded daily SpO2 measurements, and 12 (30%) adhered to daily respiratory exercises as instructed. Four participants (10%) were admitted to hospital during the follow-up period. Five participants terminated follow-up prematurely.

**Conclusions:**

Performing self-measurements of SpO2 during home isolation due to COVID-19 infection is feasible. The feasibility of performing respiratory exercises in ambulant patients is questionable and may require more motivational interventions to increase adherence.

**Trial registration:**

ClinicalTrials.gov identifier: NCT04647747.

**Supplementary Information:**

The online version contains supplementary material available at 10.1186/s40814-023-01415-x.

## Key messages regarding feasibility

- **What uncertainties existed regarding the feasibility?**
  Uncertainties in terms of feasibility mainly centered on whether home-isolated COVID-19 patients would adhere to performing daily measurements and respiratory exercises. We wanted to explore whether patients would perform both the measurements and the respiratory exercises only based on written instruction and a brief demonstration upon inclusion. In this pilot study, researchers only interacted with participants twice—at the time of inclusion and thereafter only as needed (such as in the case of decreasing oxygen levels or worsening dyspnea).
- **What are the key feasibility findings?**
  There are two key feasibility findings. The first is that self-measurement of SpO2 is feasible in a home-isolated COVID-19-positive patient population with minimal motivational effort from the investigators. The second key feasibility finding is that performing sessions of respiratory exercises every day during a 14-day follow-up time in home-isolated COVID-19 patients may not be feasible without external motivation.
- **What are the implications of the feasibility findings for the design of the main study?**
  The key takeaways are that self-monitoring of SpO2 can be carried out by home-isolated COVID-19 patients with minimal interaction from investigators, and that sustaining motivation for respiratory exercises may be necessary during follow-up.
- **What uncertainties remain?**
  We included self-measurement of SpO2, PEF, and dyspnea as well as performance of respiratory exercises. It is uncertain if adherence to a program of respiratory exercises would have been increased if PEF and dyspnea had been excluded.

## Background

The coronavirus disease 2019 (COVID-19) is caused by the severe acute respiratory syndrome-related coronarvirus-2 (SARS-CoV-2) with severity varying from asymptomatic to multi-organ failure [1]. Respiratory symptoms are among the most common in symptomatic COVID-19 patients, ranging from mild cough to severe pneumonias [2]. Most COVID-19-positive patients exhibit only mild symptoms [2], and only 4% of confirmed COVID-19 patients in Norway were hospitalized as of the time of this study [3]. As of April 2023, there have been reported 6,896,788 deaths due to COVID-19 and 762,739,900 cases of COVID-19 worldwide [4]. Ongoing hospitalizations are still a concern as 63,376 hospitalizations due to COVID-19 were reported worldwide in March 2023 [4].

At the time of this study, 2020–2021, most SARS-CoV-2 PCR tests performed in Norway were performed in dedicated test centers [5] without any structured clinical assessment. All individuals with symptoms of COVID-19 infection and also individuals exposed to confirmed SARS-CoV-2-positive individuals were asked to perform tests. As SARS-CoV-2 is highly contagious [6], it is desirable to isolate infected individuals and avoid hospitalization when possible, to avoid overloading hospitals and reduce risk of inhospital transmission. In most of Norway, individuals with a positive test are contacted by phone to provide information about the test results and instruction concerning quarantine regulation and home isolation. The duration of home isolation in Norway at the time of this study was 6 days after symptom onset. There is no formalized procedure regarding follow-up of COVID-19 patients. The safety aspects and outcomes of home isolation are, however, unknown. This is especially relevant in COVID-19 due to the phenomenon of “silent hypoxemia” [7, 8], which may result in severely ill patients without a corresponding subjective feeling of illness. Patients with silent hypoxemia may progress into a more serious respiratory failure [7]. This makes home isolation more problematic, as it is difficult to differentiate between those who would develop a more serious illness (including silent hypoxemia) and those who would not. To deliver effective and safe care for the majority of patients with COVID-19, novel approaches are needed [9].

One potential way to ensure sufficient safety measures is through in-home observation of the infected individual. Peripheral oxygen saturation (SpO2) is a quick and easily measurable way of assessing respiratory function. Furthermore, early mobilization is recommended as part of treatment for hospitalized patients with pneumonias, with some evidence of effect during the acute infective phase, mainly on reduced length of stay [10, 11]. Respiratory exercises may be a valuable treatment for COVID-19 patients and has the potential to reduce the number of individuals admitted to hospitals. However, while at home, patients with COVID-19 will not have the usual access to a respiratory physiotherapist both because of lack of resources and because of the risk of infecting others. Protocols focusing on labor-intensive tele-rehabilitation have been developed [12, 13], but to our knowledge, no study has examined the effect of brief self-managed treatment interventions with more realistic follow-up support, taking the resource scarcity of a pandemic into consideration.

To assess whether home-based respiratory exercises and monitoring can lower hospitalization and morbidity rates in COVID-19 patients, a randomized controlled trial must be conducted. However, first, it is crucial to confirm the feasibility of self-monitoring and self-managed respiratory exercises in individuals who are isolated at home due to the virus. The two main aims of this study were therefore to assess the feasibility of out-of-hospital, home-isolated COVID-19 patients conducting self-monitoring of respiratory function and symptoms, as well as performing respiratory exercises with a minimum of instruction and follow-up.

## Methods

### Study design

The study was designed as a single-arm prospective trial to assess the feasibility of self-measurements of SpO2 and performance of respiratory exercises. The number of participants was predetermined to be 40. This was based on previous recommendations of sample sizes of 30 to 50 participants [14, 15]. The study was managed by Østfold Hospital Trust, Norway, which has a catchment area of 317,000 residents [16]. Patients were recruited between December 2020 and February 2021. All Norwegian residents with either symptoms of possible COVID-19 (e.g., fever, cough, dyspnea, anosmia) or close contact were encouraged to perform a COVID-19 test at the time of this study. Most tests were performed at dedicated test centers or at emergency primary health care centers, meaning outside of the hospital, but all tests were analyzed in the microbiology laboratory at Østfold Hospital Trust.

The study was pre-registered at ClinicalTrials.gov under the identifier NCT04647747. The Regional Committees for Medical Research Ethics Southeast Norway approved the trial before it started, under the identifier REK 172708.

### Recruitment

A list of positive test results analyzed during the last 24 h was generated by the laboratory and provided to a member of the project staff. A new list was provided each weekday. Inpatients with positive tests and patients under the age of 18 were excluded. Based on the capacity of the project staff, one or two participants were randomly chosen from the list and contacted by telephone. There was no structured selection process. The participants received brief information on COVID-19, the phenomenon silent hypoxemia, and how silent hypoxemia was seen as a possible harbinger of more serious illness. Participants willing to participate in the study were sent an e-mail, including study information, a project diary, and informed consent forms. The study information included background information regarding COVID-19 and rationale of the study, how to perform respiratory exercises, how to perform measurements, and how to act on different results from the measurements. The project diary was a booklet in which participants had to complete data regarding measurements and whether respiratory exercises were completed and, if so, the duration of the exercises.

The following day, a member of the study personnel visited the participant(s). Practical instructions on how to use the equipment for objective measurements and how to perform the respiratory exercises were given. Participants received the measurement equipment and a paper version of the project diary. Written consent forms were collected, and blood samples for biobanking were drawn.

### Procedures

The participants were followed for 14 days. During the follow-up period, participants were instructed to measure their SpO2, peak expiratory flow (PEF), and rate their subjective grading of dyspnea four times a day and note the results in the provided diary. Additionally, they were asked to perform respiratory exercises twice a day. During the follow-up period, there was no contact made with the participants except at the time of inclusion and after the conclusion of the 14-day follow-up period. The participants were instructed to contact the hospital only under specific circumstances, which are described below. Upon completing the 14-day follow-up period, the participants were requested to return the study equipment, submit a completed project diary, and provide additional blood samples. The two sets of blood samples were obtained and preserved for biobanking with the aim of using them for future research within this population. These samples were not utilized in the present study.

### Measurements

SpO2 was measured using a peripheral pulse oximeter (Nellcor™ Portable SpO2 Patient Monitoring System PM10N, Covidien, Minneapolis, USA) applied on a finger with good circulation and free of nail polish and scored on a 9-step numeric rating scale (NRS) from 100 to 92 or below. A reference to actions needed was attached to the score 92 or below. PEF was measured using a PEF meter (Mini-Wright Standard Peak Flow Meter, Clement Clark International Ltd., Essex, England), with possible scores ranging between 60 and 880 L/min. The degree of dyspnea was measured using a 0–10 numeric rating scale (NRS) with anchors no shortness of breath (zero–0) and shortness of breath as bad as can be (ten–10). This is a validated scale used to assess dyspnea [17]. Participants reported all measurements in the project diary.

### Respiratory exercises

Patients were given instructions, both written (text and figures) and once practically by a member of the project staff, for light aerobic exercises, breathing, and cough control, including general advice to maintain some activity. The exercises consisted of shoulder rolls, arms extended overhead, squats, and sit-to-stand, information about sitting positions for breathing control, use of prone positioning, pursed lip breathing, and controlled coughing technique. This was based on a standard inhospital treatment guideline adapted for ease of use at home and without follow-up or monitoring by physiotherapists.

### Actions taken based on SpO2 and dyspnea rating

If, at any time during follow-up, SpO2 fell below 93% or NRS increased above 4, participants were instructed to perform an additional session of respiratory exercises. After completing the exercises, they were instructed to rest for approximately 5 min before performing a new measurement. If the repeat SpO2 was below 91% or NRS was above 4, the participants were instructed to contact the hospital to consider admission.

### Analysis

In this pilot study, we tested no formal hypothesis. Continuous variables are expressed by mean and standard deviation (SD) or median and interquartile range (IQR), whereas categorical variables are expressed by percent. The primary feasible outcomes are expressed as percent and 95% confidence interval (CI) calculated via Wilson score interval.

The primary feasibility outcomes were expressed by the proportion of participants who completed self-measurements of SpO2 and the proportion who performed respiratory exercises daily for the entire follow-up time or until hospital admission. We considered self-measurements of SpO2 and performance of respiratory exercises, performed for at least 5 min per session, feasible if performed every day during follow-up by at least 80% of participants [18].

Participants who terminated registrations prematurely were included in the analyses up until day 14. Participants who were hospitalized were included to the day of hospitalization only.

## Results

Through 13 weeks, 40 participants were included, 17 males and 23 females. Participant baseline characteristics can be found in Table 1.

**Table 1:** Participant baseline characteristics

Age in years, mean (SD)	52 (10)
Women, *n* (%)	23 (58%)
Asymptomatic^a^, *n* (%)	6 (15%)
Symptoms, *n* (%)	34 (85%)
Muscle pain	9 (23%)
Fatigue	9 (23%)
Nasal congestion	4 (10%)
Fever	9 (23%)
Cough	11 (28%)
Sore throat	11 (28%)
Nausea/vomiting	2 (5%)
Agnosia	2 (5%)
Symptom debut prior to inclusion in days^b^, mean (SD)	4.6 (1.9)

^a^No symptoms prior to testing

^b^In patients with symptoms as opposed to exposure only

**Fig. 1:**
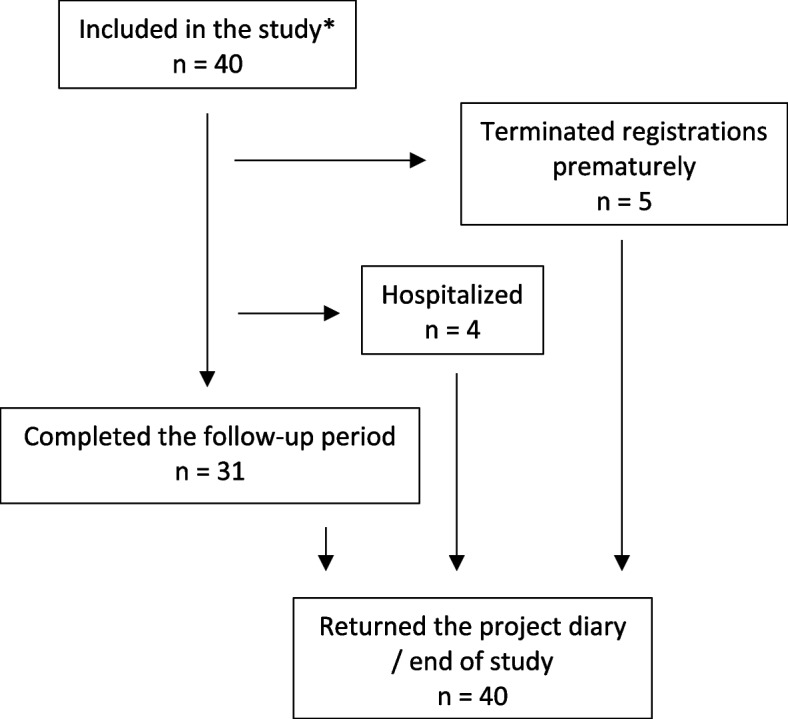
Flowchart. *The total number of patients contacted by telephone was not recorded

Out of the total participants, 31 (78%) completed the diaries for the full follow-up period (14 days), four (10%) were hospitalized, and five (12.5%) ended registrations prematurely (Fig. 1). Two hospitalizations were due to a decrease in general condition, while the other two were due to worsening dyspnea. The admissions took place on days 4, 5, 9, and 10 following inclusion. Participants who prematurely terminated registrations did so on days 4, 5, 8, 9, and 12 after inclusion, with reasons for early termination not being explored. All participants, including those who terminated registrations prematurely, returned the project diaries. Initial and follow-up blood samples for biobanking were acquired from 39 and 37 participants respectively.

### Primary feasibility outcomes

Daily measurements of SpO2 were performed by 35 participants (87.5% *CI* 73.9%, 94.5%). At least one daily session of respiratory exercises lasting for more than 5 min was performed by 12 participants (30% *CI* 18.1%, 45.4%). Mean (SD) duration of the sessions performed by these 12 participants was 13.3 (5.6) min.

### Other outcomes

Mean (SD) SpO2 measurements were 3.7 (0.8) times daily. Mean (SD) SpO2 was 97.5 (1.3%). No cases of silent hypoxia were detected.

Three participants performed all sessions of respiratory exercises (two daily sessions, 28 sessions in total), while four participants did not perform any session. Median (IQR) number of performed sessions of respiratory exercises was 20.5 (5-24). Median (IQR) number of days where respiratory exercises were performed was 12 (4-14) of a possible total of 14 days. If all participants are included (both those who performed exercises daily and those who did not), the mean duration of each session of respiratory exercises was 12.0 (5.4) min. There were no differences in compliance in measuring SpO2 between those who performed respiratory exercises and those who did not.

Daily measurements of PEF and NRS were performed by 33 (82.5%) and 31 (77.5%) participants, respectively. Mean (SD) PEF and NRS were 424 (114) L/min and 2.6 (1.1), respectively.

## Discussion

The main objective in this study was to assess the feasibility of COVID-19 patients performing self-monitoring of SpO2 and performing respiratory exercises with minimal instruction and external motivation. Feasibility criteria were met for SpO2 measurements, but not for respiratory exercises.

No cases of silent hypoxemia were found. However, the sample size in this study was relatively small; therefore, no definite conclusion can be made based on our results. One of the hospitalized participants had a decrease in SpO2 (92%), but it was accompanied by a corresponding increase in dyspnea.

We did not find any parameters that differed between the hospitalized and the nonhospitalized participants. A larger sample size will be needed to reveal any factors that may lead to early identification of patients who will be in need for hospitalization. There were no formal assessment criteria for general condition.

According to our criteria for self-measurement, we considered self-measuring SpO2 feasible if > 80% of included participants performed at least one SpO2 measurement daily during a 14-day follow-up. Participants measured SpO2 several times (mean 3.7 times) each day during follow-up. This indicates that no further motivational intervention is required for home-isolated COVID-19-positive individuals to perform frequent and regular SpO2 measurements.

A similar study was conducted by Wilcock et al. [19] who included patients diagnosed with COVID-19 within the last 7 days, where patients were provided with a pulse oximeter and a symptom and oximetry diary. Participants measured SpO2 twice daily and recorded the degree of breathlessness and symptoms. They were asked to perform measurements for 14 days or until complete recovery, whichever came first. Participants were to contact healthcare providers in case of a significant decrease in SpO2 or in general condition. One of the secondary outcomes was the diary completion rate, which was 79% (41/52 participants returned the diary). The number of participants performing daily SpO2 measurements was not noted. Other studies reporting high compliance with self-measuring SpO2 also performed frequent, mostly daily, reminders or patient contacts which make comparison to our study difficult [20–22].

Adherence to a program of daily respiratory exercises while in home isolation during active COVID-19 infection was not feasible according to our feasibility criteria. Additional motivational efforts may be needed to increase adherence. Our predefined criterium for feasibility was that at least 80% of participants were to perform at least one session of respiratory exercises daily during the entire follow-up, whereas only 30% of the participants performed daily sessions of respiratory exercises. However, the median total number of performed sessions was 20.5, which constitutes 73% of the total number (*n* = 28) of planned sessions of respiratory exercises during the follow-up time. Whether daily consistency in exercise performance or total volume of exercises during active illness is of greatest importance remains unclear. Nevertheless, future research on respiratory exercises in COVID-19 or other respiratory infections may consider focusing on the total volume of exercises rather than performing exercises every day.

A larger study population is needed to explore the effect of respiratory exercises on an out-of-hospital patient population.

Self-measurement of dyspnea was included in the study to be able to diagnose silent hypoxia, in contradiction to symptomatic hypoxia. It was measured daily during follow-up by 77.5% of the participants. It should also be noted that if a subjective symptom grading is to be used for assessing whether a patient should be hospitalized or not, the absolute NRS scores should be interpreted with caution as the answers may be biased depending on whether the participant hopes for hospitalization or not. The mean degree of dyspnea was relatively low at 2.6 of 10. The usefulness of self-registration of dyspnea is probably limited as the SpO2 is probably of more importance to detect clinical worsening. We would not recommend including it in future studies unless subjective grading of dyspnea is of particular interest to the researchers. This is to limit the number of self-registrations in order to maximize adherence.

Self-measurement of PEF was included in this pilot for exploratory reasons. No decisions were dependent on the results of the PEF measurements. The participants were requested to measure PEF four times a day, like the other measurements. However, they were instructed to take only one measurement at each of the four designated times, unlike the usual recommendation of three measurements. This methodology may have resulted in a systematic bias towards lower values. Even though self-measurements of PEF could be considered feasible (82.5% adherence), the benefit of including PEF is unclear. As discussed above, it may be beneficial to avoid any excess self-measurements to maximize adherence, and we therefore recommend avoiding it in future research unless PEF is of particular interest for the researchers.

The total number of telephone contacts made to recruit participants was unfortunately not documented. Furthermore, the absence of a formal selection procedure may have resulted in a selection bias to some degree. The reasons for early termination by five of the participants was unfortunately not explored, as this could be of value to plan inclusion criteria for future studies. Future research should incorporate the participants’ prior medical history, which was not registered in the current study. It should also be noted that this study was carried out at a time when COVID-19 was covered in media to a large extent, and many feared the consequences of the disease. This might have contributed to a higher degree of adherence than what could normally be expected. The pulse oximeters provided were relatively expensive hospital-grade devices, which may have increased compliance more than cheaper self-bought pulse oximeters. The accuracy of measurements and performance of respiratory exercises are uncertain as these were self-completed by the participants without any external control.

The two main strengths of this study were that participants were included within 48 h from positive test result, and the methodology of this study is applicable to clinical practice. A minimum of resources was used during the study.

## Conclusion

Performing self-measurements of SpO2 during home isolation due to COVID-19 infection is feasible, whereas the feasibility of performing respiratory exercises is questionable and may require more motivational interventions to increase adherence during the follow-up time. The results of this pilot can be used to guide future research in home-isolated or other out-of-hospital patient populations.

### Supplementary Information

**Additional file 1. **

**Additional file 2. **

**Additional file 3. **

**Additional file 4. **

## Data Availability

The datasets used and/or analyzed during the current study are available from the corresponding author on reasonable request.

## Abbreviations

COVID-19: Coronavirus disease 2019
SARS-CoV-2: Severe acute respiratory syndrome-related coronavirus-2
SpO2: Peripheral oxygen saturation
NRS: Numeric rating scale
PEF: Peak expiratory flow
REK: Regional ethical committee
SD: Standard deviation
IQR: Interquartile range
