# Supplementary material for: The feasibility of self-performing measurements of peripheral oxygen saturation and respiratory exercises in home-isolated COVID-19 patients—a single-arm prospective trial
Source: Pilot Feasibility Stud. 2023 Dec 2;9:195. doi: 10.1186/s40814-023-01415-x (PMC10693052; doi:10.1186/s40814-023-01415-x)
Supplement: Supplementary file 1 — Additional file 1. [file 40814_2023_1415_MOESM1_ESM.pdf]

SYKEHUSET ØSTFOLD

FORESPØRSEL OM Å AVGI BIOLOGISK MATERIALE TIL

FORSKNINGSBIOBANK FOR COVID-19

BAKGRUNN OG HENSIKT

Formålet med biobanken er å samle biologisk materiale fra personer som har testet positivt for koronaviruset SARS-CoV-2 til bruk i nåværende og fremtidig helseforskning. Både pasienter som er innlagt ved Sykehuset Østfold for behandling av covid-19 og andre som har testet positivt for viruset vil kunne bli spurt om å avgi materiale til den generelle biobanken og om samtykke til at relevante helseopplysninger kan benyttes til forskning. Dette vil legge til rette for nødvendig forskning som vil kunne bidra til bekjempelse av viruset og gi kunnskap som kan forbedre utredning, behandling og oppfølging av pasienter med denne sykdommen.

Sykehuset Østfold HF er ansvarlig for biobanken og tilhørende register. Det biologiske materialet og opplysningene om deg blir oppbevart på ubestemt tid og skal brukes i fremtidig forskning på covid-19.

HVILKET BIOLOGISK MATERIALE SKAL INNSAMLES?

For pasienter som er innlagt med covid-19 på sykehuset vil det tas ekstra blodprøver, luftveisprøve, urinprøve og avføringsprøve. Disse prøvene vil som regel bli tatt samtidig med andre prøver som tas i forbindelse med behandlingen på sykehuset. Blodprøver innebærer et ubehag i form av et stikk. Luftveisprøver fra hals eller nese vil også kunne medføre ubehag når prøven tas.

Fra deltakere som ikke er innlagt på sykehus vil det være aktuelt å samle blodprøver, luftveisprøve, urinprøve og avføringsprøve. Deltakere vil bli kontaktet av studiepersonell for å avtale tid og sted for prøvetaking.

Prøvetakingen medfører ingen betydelig risiko.

BREDT SAMTYKKE

Når du avgir biologisk materiale til denne generelle forskningsbiobanken, avgir du også et bredt samtykke til at materiale og relevante helseopplysninger kan brukes til fremtidig forskning innen covid-19.

Forskningsprosjekter som skal bruke data og innsamlete prøver vil bli vurdert av regional komité for medisinsk og helsefaglig forskningsetikk, som må godkjenne at studien dekkes av det avgitte samtykket.

INNSAMLING OG BRUK AV HELSEOPPLYSNINGER

Biobanken vil inneholde noen opplysninger om deg, som for eksempel navn, fødselsnummer, diagnose og behandlingssted. Denne informasjonen er lagret i et elektronisk register som bare er tilgjengelig for ansatte ved sykehuset som jobber med biobanken. Alt fysisk materiale lagres uten identifiserende kjennetegn. Prøvegiver kan bare identifiseres gjennom en koblingsnøkkel som skal beskytte din identitet, men samtidig gjør det mulig å knytte opplysningene om deg til ditt materiale gjennom en kodeliste. Sykehuset Østfold HF er ansvarlig for at koblingsnøkkel oppbevares og forvaltes forsvarlig. Materiale og opplysningene om deg lagres permanent og vil analyseres i forbindelse med spesifiserte forskningsprosjekter.

Det lagres også informasjon om pasienter som er innlagt med covid-19 ved Sykehuset Østfold i et internt register som har til hensikt å kvalitetssikre behandlingen og oppfølgingen av pasienter ved sykehuset. Ved samtykke til deltakelse i prosjektet samtykker du også til at disse opplysningene kan benyttes til forskning. I tillegg til relevante helseopplysninger ber vi også om samtykke til å registrere informasjon om etnisk bakgrunn.

Ved eventuell publisering av forskningsresultater som benytter materiale fra biobanken vil resultatene presenteres slik at identiteten til de inkluderte pasientene ikke kommer frem.

SAMMENSTILLING AV DATA FRA BIOBANKEN MED ANDRE OPPLYSNINGER

I enkelte forskningsprosjekter kan det være aktuelt å sammenstille informasjon fra biobanken og tilhørende register med opplysninger fra din pasientjournal inkludert blodprøveanalyser og bildediagnostikk, interne kvalitetsregistre, helseundersøkelser, helseregistre eller offentlige administrative registre, som for eksempel Nasjonalt vaksinasjonsregister, Norsk pasientregister, Reseptregisteret, Dødsårsaksregisteret, med flere.

GENETISKE UNDERSØKELSER

Det kan være aktuelt å gjøre genetiske analyser av det materialet som er samlet inn. Disse genetiske analysene har som mål å finne eventuelle sammenhenger mellom arvestoffet og smitteforekomst, sykdomsutvikling og respons på terapi.

Dersom materialet du har avgitt skal benyttes i prosjekter der det gjøres genetiske undersøkelser som kan ha betydning for din helse, vil du kunne bli kontaktet i forkant av prosjektet med tilbud om informasjon, oppfølging og veiledning. Det kan også være nødvendig å be om et nytt samtykke til slike studier.

GODKJENNING AV FREMTIDIGE FORSKNINGSPROSJEKTER

Alle fremtidige forskningsprosjekter som benytter materialet fra deg skal forhåndsgodkjennes av en regional komité for medisinsk og helsefaglig forskningsetikk.

INFORMASJON OM FREMTIDIGE PROSJEKTER

Som deltaker i den generelle forskningsbiobanken ved Sykehuset Østfold har du krav på informasjon om prosjektene som benytter materiale fra biobanken. Dersom du ønsker vil du regelmessig få tilsendt et nyhetsbrev med informasjon om dette. Informasjonen kan du også finne på Sykehuset Østfolds internettside www.sykehuset-ostfold.no under Fag – Forskning og innovasjon – Forskningsbiobank.

UTLEVERING AV PRØVEMATERIALE

Det kan være aktuelt at biologisk materiale og opplysninger om deg utleveres til forskningsinstitusjoner i inn- og utland som ledd i forskningssamarbeid og publisering. Dette kan være land med lover som ikke tilfredsstiller europeisk personvernlovgivning. Materialet vil kun utleveres uten navn, fødselsnummer eller andre direkte gjenkjennende opplysninger, og prosjektleder vil sikre at dine opplysninger blir ivaretatt på en trygg måte.

DET ER FRIVILLIG Å DELTA

Å avgi biologisk materiale til Sykehuset Østfolds forskningsbiobank for covid-19 er frivillig og krever samtykke. Det vil ikke ha noen betydning for din behandling dersom du ikke ønsker å avgi prøve, eller dersom du senere ønsker å trekke deg fra prosjektet.

MULIGHET FOR Å TREKKE SITT SAMTYKKE, INNSYNSRETT, ENDRING OG SLETTING AV OPPLYSNINGER

Du kan til enhver tid få innsyn i hvilket materiale som er lagret fra deg. Du kan når som helst kreve at materialet blir destruert, uten at du må oppgi noen grunn. Destruksjon av materialet vil imidlertid ikke innebære sletting av utledete opplysninger som har inngått i sammenstilling eller analyser.

Forskningsbiobanken er godkjent av Regional komité for medisinsk og helsefaglig forskningsetikk (#132065).

Etter ny personopplysningslov har behandlingsansvarlig Sykehuset Østfold og ansvarlig for biobanken, Waleed Ghanima, et selvstendig ansvar for å sikre at behandlingen av dine opplysninger har et lovlig grunnlag. Dette prosjektet har rettslig grunnlag i EUs personvernforordning artikkel 6 og 9.

Du har rett til å klage på behandlingen av dine opplysninger til Datatilsynet.

KONTAKT

Ansvarlig og kontaktperson for biobanken er forskningssjef Waleed Ghanima, som kan kontaktes på telefon 69 86 00 00 eller e-post: waleed.ghanima@so-hf.no.

Du kan ta kontakt med institusjonens personvernombud dersom du har spørsmål om behandlingen av dine personopplysninger i prosjektet. Sykehuset Østfolds personvernombud kan kontaktes på telefonnummer 69 86 00 00, eller via e-post til personvernombudet@so-hf.no.

SAMTYKKE TIL LAGRING AV BIOLOGISK MATERIALE

Jeg er villig til å avgi bredt samtykke til at mitt biologiske materiale og relevante helseopplysninger kan oppbevares varig i Sykehuset Østfolds forskningsbiobank for covid-19 og bli benyttet i fremtidig forskning.

Sted og dato   Deltakers signatur

Deltakers navn med trykte bokstaver

NYHETSBREV

Ønsker du å motta nyhetsbrev om forskningsprosjekter som bruker materiale fra Sykehuset Østfolds forskningsbiobank for covid-19?

☐ Ja, jeg ønsker å få nyhetsbrev tilsendt som brevpost til folkeregistrert adresse.

☐ Ja, jeg ønsker å få nyhetsbrev tilsendt elektronisk til følgende e-postadresse: ________________________

☐ Nei, jeg ønsker ikke å få tilsendt nyhetsbrev.
