# Supplementary material for: The feasibility of self-performing measurements of peripheral oxygen saturation and respiratory exercises in home-isolated COVID-19 patients—a single-arm prospective trial
Source: Pilot Feasibility Stud. 2023 Dec 2;9:195. doi: 10.1186/s40814-023-01415-x (PMC10693052; doi:10.1186/s40814-023-01415-x)
Supplement: Supplementary file 2 — Additional file 2. [file 40814_2023_1415_MOESM2_ESM.pdf]

SYKEHUSET ØSTFOLD

VIL DU DELTA I FORSKNINGSPROSJEKTET SHYCOV – HJEMMEMONITORERING AV COVID-19 PASIENTER?

FORMÅLET MED PROSJEKTET OG HVORFOR DU BLIR SPURT

Dette er et spørsmål til deg om å delta i et forskningsprosjekt for å undersøke om bruk av oksygenmåler og enkle fysioterapiøvelser kan redusere faren for alvorlig forløp av covid-19 hos personer som er smittet med koronavirus SARS-CoV-2. Du blir invitert til å delta i prosjektet fordi Sykehuset Østfold har registrert at du har avlagt en positiv test for koronavirus SARS-CoV-2.

HVA INNEBÆRER PROSJEKTET FOR DEG?

Deltakelse i prosjektet innebærer at du registrerer observasjoner om sykdomsutvikling hjemmefra. Du skal ikke komme til sykehuset for å delta i prosjektet, men dersom din tilstand forverres skal du selvfølgelig ta kontakt, ut ifra målingene som gjøres. Dette står nærmere beskrevet i eget skriv.

Observasjonsperioden varer i to uker. I denne perioden skal du gjennomføre målinger med en tilsendt oksygenmåler, og en pustekraftmåler, enkle fysioterapiøvelser og registrering av data i en prosjektdagbok.

Måling av oksygennivået i blodet gjøres ved gjennomlysning av fingeren med en oksygenmåler. Avhengig av verdien som avleses kan du bli bedt om å foreta deg ulike handlinger, som gjentatt måling, fysioterapiøvelser, eller å kontakte sykehuset.

I prosjektet er det også utarbeidet et fysioterapiprogram med enkle øvelser, som du oppfordres til å gjennomføre dersom du føler deg opplagt nok til det. Fysioterapidelen inneholder også råd om liggestilling.

Observasjoner og eventuelle fysioterapiøvelser gjennomføres fire ganger daglig (kl. 8, 12, 16 og 20), og resultatet føres i vedlagte prosjektdagbok. I prosjektdagboken registreres også opplysninger om andre forkjølelsessymptomer. Hver observasjon kan ta inntil 30 minutter, avhengig av antall målinger som gjøres og om man gjennomfører fysioterapiøvelser.

Detaljer om oksygenmålingen, fysioterapiøvelsene og registering finnes i vedlagte informasjonsmateriell. Vi vil i løpet av observasjonsperioden kontakte deg to ganger for å høre hvordan det går med registreringen. Ved spørsmål kan du også ta kontakt med oss (se kontaktinformasjon på siste side).

Opplysningene som registreres i prosjektdagboken vil bli lagret ved Sykehuset Østfold når observasjonsperioden avsluttes. Det vil også bli aktuelt å sammenstille opplysningene med relevante opplysninger i din pasientjournal ved sykehuset.

MULIGE FORDELER OG ULEMPER

En mulig fordel med å delta i prosjektet er at hyppige oksygenmålinger kan gjøre at en eventuell sykdomsforverring oppdages tidligere, og at man dermed også tidligere får vurdert behov for sykehusinnleggelse.

En annen fordel er at man får tilgang til et program med fysioterapiøvelser som kan ha positiv effekt på sykdomsforløpet.

Det er ingen vesentlige ulemper ved deltakelse utover tiden som kreves for å gjennomføre øvelser og observasjoner.

FRIVILLIG DELTAKELSE OG MULIGHET FOR Å TREKKE DITT SAMTYKKE

Det er frivillig å delta i prosjektet. Dersom du ønsker å delta, undertegner du samtykkeerklæringen på siste side. Du kan når som helst og uten å oppgi noen grunn trekke ditt samtykke. Det vil ikke ha noen negative konsekvenser for deg eller din behandling hvis du ikke vil delta eller senere velger å trekke deg. Dersom du trekker tilbake samtykket, vil det ikke forskes videre på dine helseopplysninger. Du kan også kreve at dine helseopplysninger i prosjektet slettes eller utleveres innen 30 dager. Adgangen til å kreve destruksjon, sletting eller utlevering gjelder ikke dersom materialet eller opplysningene er anonymisert. Denne adgangen kan også begrenses dersom opplysningene er inngått i utførte analyser, eller dersom materialet er bearbeidet og inngår i et annet biologisk produkt.

Dersom du senere ønsker å trekke deg eller har spørsmål til prosjektet, kan du kontakte prosjektleder (se kontaktinformasjon på siste side).

HVA SKJER MED OPPLYSNINGENE OM DEG?

Opplysningene som registreres om deg skal kun brukes slik som beskrevet under formålet med prosjektet, og planlegges brukt til 2022. Eventuelle utvidelser i bruk og oppbevaringstid kan kun skje etter godkjenning fra REK og andre relevante myndigheter. Du har rett til innsyn i hvilke opplysninger som er registrert om deg og rett til å få korrigert eventuelle feil i de opplysningene som er registrert. Du har også rett til å få innsyn i sikkerhetstiltakene ved behandling av opplysningene. Du kan klage på behandlingen av dine opplysninger til Datatilsynet og institusjonen sitt personvernombud.

Alle opplysningene vil bli behandlet uten navn og fødselsnummer eller andre direkte gjenkjennende opplysninger (=kodede opplysninger). En kode knytter deg til dine opplysninger gjennom en navneliste. Det er kun prosjektmedarbeidere som har tilgang til denne listen.

Opplysningene om deg vil bli oppbevart i fem år etter prosjektslutt av kontrollhensyn.

FORSIKRING

Deltakere i dette prosjektet er forsikret i henhold til pasientskadeloven.

OPPFØLGINGSPROSJEKT

Du kan bli kontaktet på nytt med spørsmål om deltakelse i oppfølgingsprosjekter dersom dette blir aktuelt.

GODKJENNINGER

Regional komité for medisinsk og helsefaglig forskningsetikk har gjort en forskningsetisk vurdering og godkjent prosjektet i sak 172708.

I henhold til personopplysningsloven har behandlingsansvarlig Sykehuset Østfold HF og prosjektleder Waleed Ghanima et selvstendig ansvar for å sikre at behandlingen av dine opplysninger har et lovlig grunnlag. Vi behandler opplysningene basert på ditt samtykke med rettslig grunnlag i EUs personvernforordning artikkel 6 og 9.

KONTAKTOPPLYSNINGER

Dersom du har spørsmål til prosjektet eller ønsker å trekke deg fra deltakelse, kan du kontakte prosjektleder Waleed Ghanima på telefon 69 86 00 00 eller e-post waleed.ghanima@so-hf.no.

Dersom du har spørsmål om personvernet i prosjektet, kan du kontakte personvernombudet ved institusjonen på telefon 69 86 00 00 eller e-post personvernombudet@so-hf.no.

Du har rett til å klage på behandlingen av dine opplysninger til Datatilsynet, som kan kontaktes på e-post postkasse@datatilsynet.no.

SAMTYKKE TIL DELTAKELSE I FORSKNINGSPROSJEKTET SHYCOV

☐ Jeg samtykker til å delta i prosjektet og til at mine personopplysninger brukes slik det er beskrevet.

☐ Jeg samtykker til å avgi biologisk materiale og at dette lagres og benyttes til fremtidig forskning som beskrevet i informasjonsskriv om «Sykehuset Østfolds forskningsbiobank for covid-19».

Sted og dato

Deltakers signatur

Deltakers navn med trykte bokstaver
