# Supplementary material for: The feasibility of self-performing measurements of peripheral oxygen saturation and respiratory exercises in home-isolated COVID-19 patients—a single-arm prospective trial
Source: Pilot Feasibility Stud. 2023 Dec 2;9:195. doi: 10.1186/s40814-023-01415-x (PMC10693052; doi:10.1186/s40814-023-01415-x)

SYKEHUSET ØSTFOLD

SHYCOV - STUDIE OM OKSYGENMETNING OG FYSIOTERAPI VED COVID-19

FORMÅL OG GJENNOMFØRING AV PROSJEKTET

Tusen takk for at du vil være med på studien! Vi ønsker å undersøke pasienter som er smittet med coronavirus (SARS-CoV-2). Vi skal undersøke om man får redusert metning av oksygen i blodet, uten at man får noen symptomer av det. Vi ønsker også å undersøke effekten av enkel fysioterapi for å bedre pusten.

For å kunne undersøke dette er det viktig at du gjør målingene fire ganger om dagen og fyller ut prosjektdagboken (eget hefte). Du skal da måle metningen, angi hvor tungpusten du er og gjøre en pustemåling (også kalt PEF). Du skal også gjøre fysioterapi fast to ganger om dagen, og eventuelt flere ganger dersom metningen faller under et visst nivå eller graden av tungpust øker over et visst nivå. Mer om dette på de neste sidene og i prosjektdagboken.

TIMEPLAN FOR EN TYPISK DAG UNDER STUDIEN:

Kl. 0800

Måle metning, tungpust og pustekraft

(Eventuelt fysioterapi og ny måling av metning og tungpust)

Kl. 1200

Fysioterapi

Måle metning, tungpust og pustekraft

(Eventuelt ny runde fysioterapi og ny måling av metning og tungpust)

Kl. 1600

Måle metning, tungpust og pustekraft

(Eventuelt fysioterapi og ny måling av metning og tungpust)

Kl. 2000

Fysioterapi

Måle metning, tungpust og pustekraft

(Eventuelt ny runde fysioterapi og ny måling av metning og tungpust)

FYSIOTERAPIPROGRAM

Under vises øvelser du kan gjøre to ganger daglig eller når metningsmåleren viser en metning på 93 eller lavere. På denne og neste side vises råd og øvelser for pust og hoste-kontroll.

Forsøk å ta 20-30 repetisjoner av hver øvelse, 2-3 runder om du orker. Gjør heller få repetisjoner ofte enn mange repetisjoner sjeldent. Selv om du noen dager kan være uopplagt eller i dårlig form, bør du prøve å være i aktivitet.

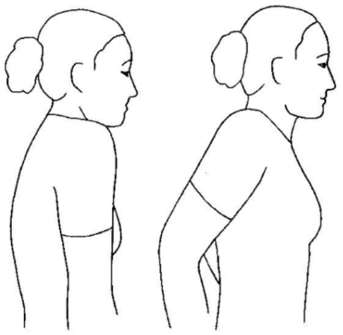

Rull på skuldrene
Stå eller sitt. Trekk skuldrene opp og trekk samtidig pusten inn, rull deretter skuldrene bakover og ned samtidig som du slipper pusten ut.

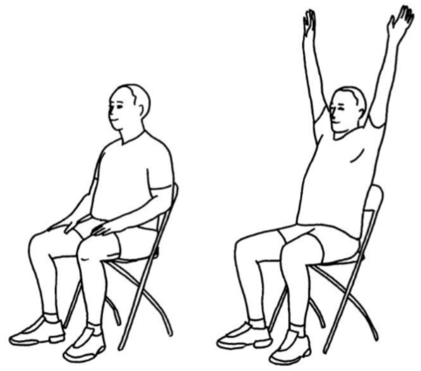

Armstrekk
Sitt på sengekanten eller en stol med armene på lårene. Før armene parallelt opp mot taket og ut til siden og tilbake til lårene, som om du former en sol.

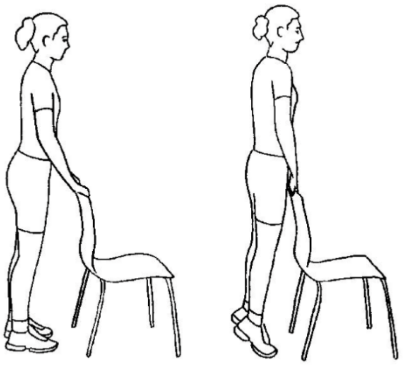

Stående tåhev med støtte
Stå bak en stol og støtt deg med hendene til stolryggen. Gå opp på tærne med begge bena og bli stående i cirka 5 sekunder. Senk deg rolig ned til stående igjen.

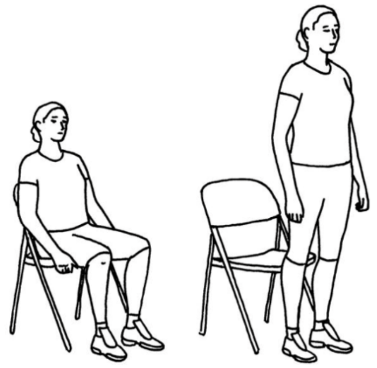

Sette og reise seg
Sitt på sengekanten eller en stol. Beveg overkroppen fremover og reis deg opp ved bruk av beina. Skyv fra med armene om nødvendig.

Her vises råd og øvelser for **pust og hoste-kontroll.**

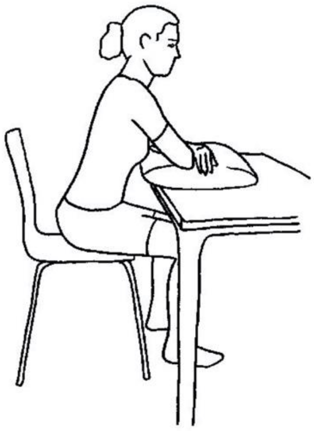

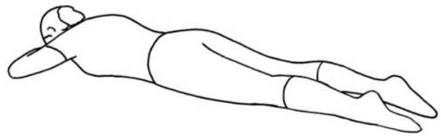

Sittende hvilestilling
Ved tungpust kan du prøve å bruke hvilestillinger. Du kan f.eks støtte deg framover et bord, legg gjerne på en pute. *Hvilestillinger kan lette pusten og du bruker mindre energi.*

Mageleie / liggende hvilestilling
Hvis du ønsker å ligge, forsøk å være så mye som mulig i mageleie. Bruke en pute under magen/ hoftene for å lettere ligge slik over tid. Mageleie har vist seg å være bra for lungefunksjonen.

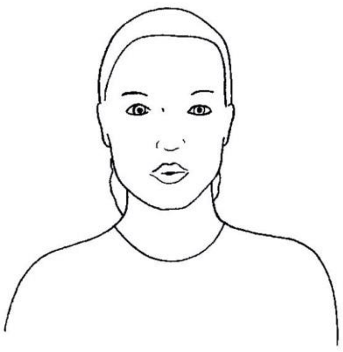

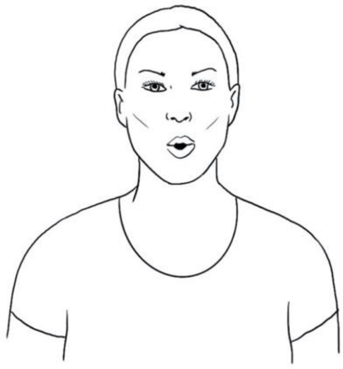

Leppepust
Pust ut med halvåpne lepper (som om du blåser på noe varmt). *Leppepust hjelper til å roe ned pusten og bidrar til å holde luftveiene åpne under utpusten. Du kan bruke denne puste-måten regelmessig ved tungpust.*

Støt-teknikk
Pust halvveis dypt inn og pust rolig ut med åpen munn som om du lager dugg på et speil. Gjenta dette 2-3 ganger. Pust så dypt inn og pust ut litt kraftigere og kortere 2-3 ganger - og avslutt med hoste om du får opp slim. *Dette er skånsomt for luftveiene og løsner og flytter på slimet.*

MÅLING AV METNING AV OKSYGEN I BLODET

Du måler metningen med metningsmåleren (se bildet under). Du må ha sittet i ro i minst fem minutter før du gjør målingen. Metningsmåleren settes på en varm finger uten neglelakk. Du leser av verdien og setter ring rundt tallet i prosjektdagboken.

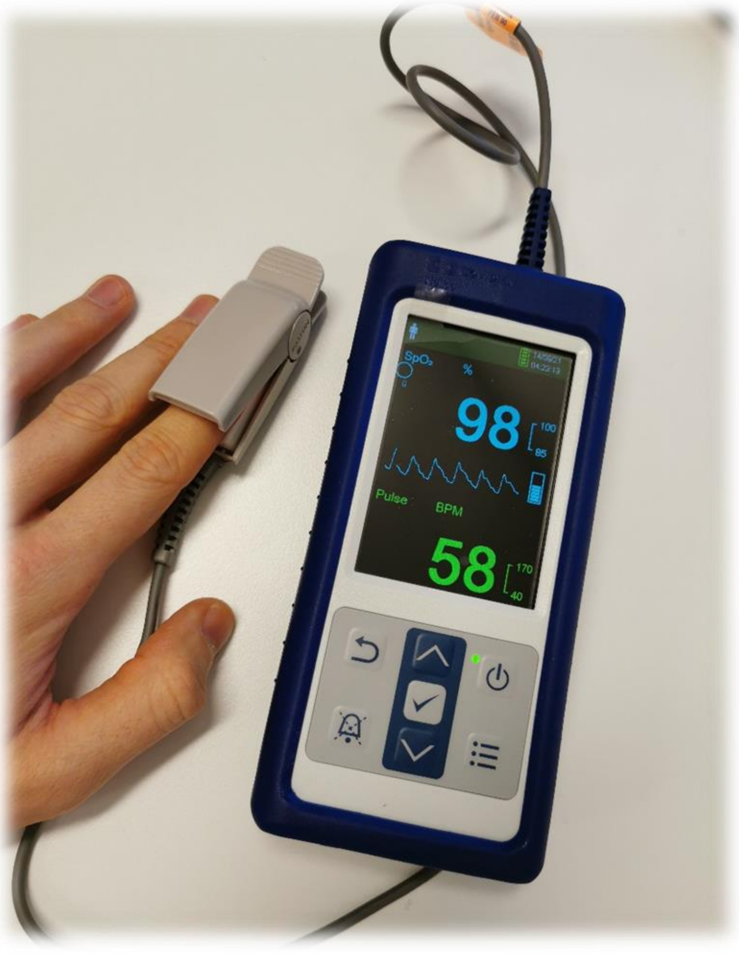

Hvis du får en måling som er <u>93 eller høyere</u>, setter du ring rundt tallet i prosjektdagboken. Du trenger da ikke gjøre ekstra fysioterapi eller kontakte sykehuset. Gå videre til måling av tungpust og pustekraft.

Hvis du får en måling som er <u>lavere enn 93</u>, så må det utelukkes at det er en feilmåling. Du må derfor gå igjennom «Gjøremålsliste ved mistanke om feilmåling av metning», se under. Hvis metningen fortsatt er lavere enn 93, skriver du tallet over de tre prikkene i prosjektdagboken og setter ring rundt tallet. Du skal deretter utføre fysioterapi (side 2-3) og måle metningen på nytt etter omtrent 10 minutter. Du skal gjøre fysioterapi selv om du kanskje allerede har gjort det før du målte. Hvis den nye metningen er 92 eller høyere setter du ring rundt tallet i prosjektdagboken, men nå under den stiplede linjen. Er den 90 eller 91, skriver du det over de tre prikkene og setter en ring rundt tallet. Hvis den nye metningen er under 90, skal du kontakte sykehuset på tlf. 698 60 000 for å vurdere om du må på sykehuset for en vurdering. Se også flytskjemaet under.

Gjøremålsliste ved mistanke om feilmåling av metning

1) Fjerne neglelakk
2) Varme hånden/fingrene
3) Plasser metningsmåleren på den andre hånden, pass på at armen er under hjertehøyde
4) Skru opp belysningen eller gå inn i et annet rom med bedre belysning

FLYTSKJEMA FOR MÅLING AV METNING

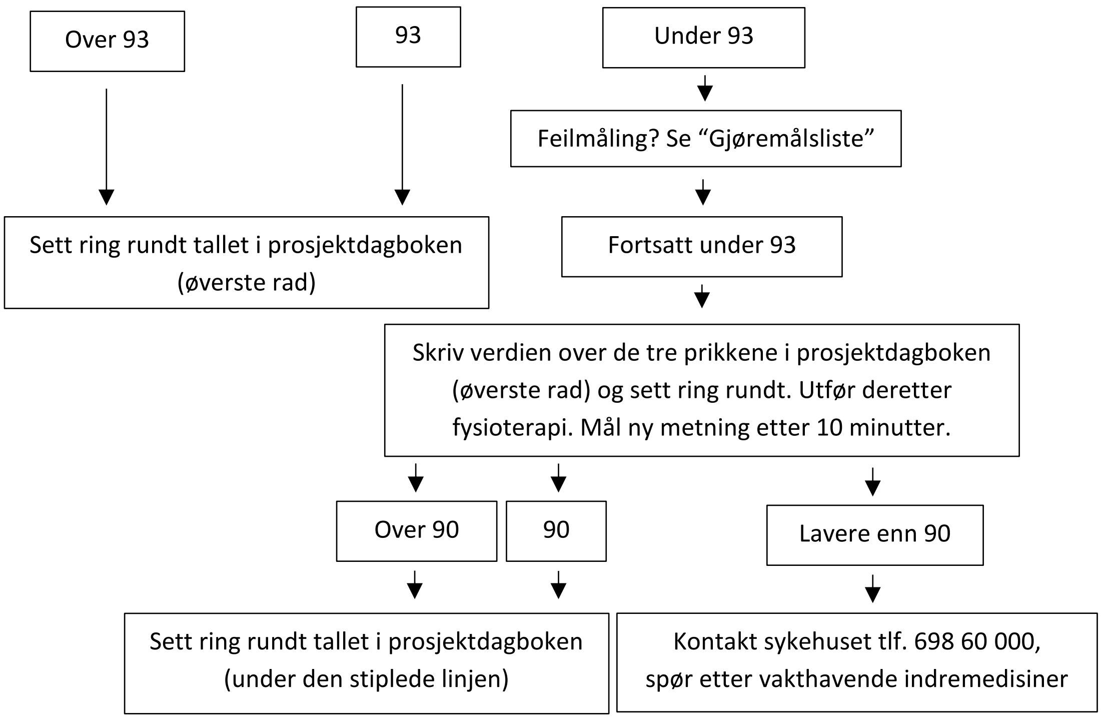

MÅLING AV TUNGPUST

Etter du har målt metningen, angir du graden av tungpust på en skala fra 0 til 10. På skalaen er 0 ingen tungpust i det hele tatt, 10 er så tungpusten det går an å bli. Sett ring rundt tallet i prosjektdagboken.

Hvis du har tungpust 0 til 4, setter du ring rundt tallet i prosjektdagboken. Du trenger da ikke gjøre noe ekstra fysioterapi og du trenger heller ikke å kontakte sykehuset.

Hvis du har tungpust 5 til 10, setter du ring rundt tallet i prosjektdagboken. Du skal deretter utføre fysioterapi (forrige side) og angi tungpust på nytt etter omtrent 10 minutter. Du skal gjøre fysioterapi selv om du kanskje allerede har gjort det. Hvis du nå har tungpust 0 til 4 setter du ring rundt tallet i prosjektdagboken, men nå under den stiplede linjen. Hvis du fortsatt har tungpust 5 til 10, skal du kontakte sykehuset på tlf. 698 60 000 for å vurdere om du må på sykehuset for en vurdering. Se også flytskjemaet under.

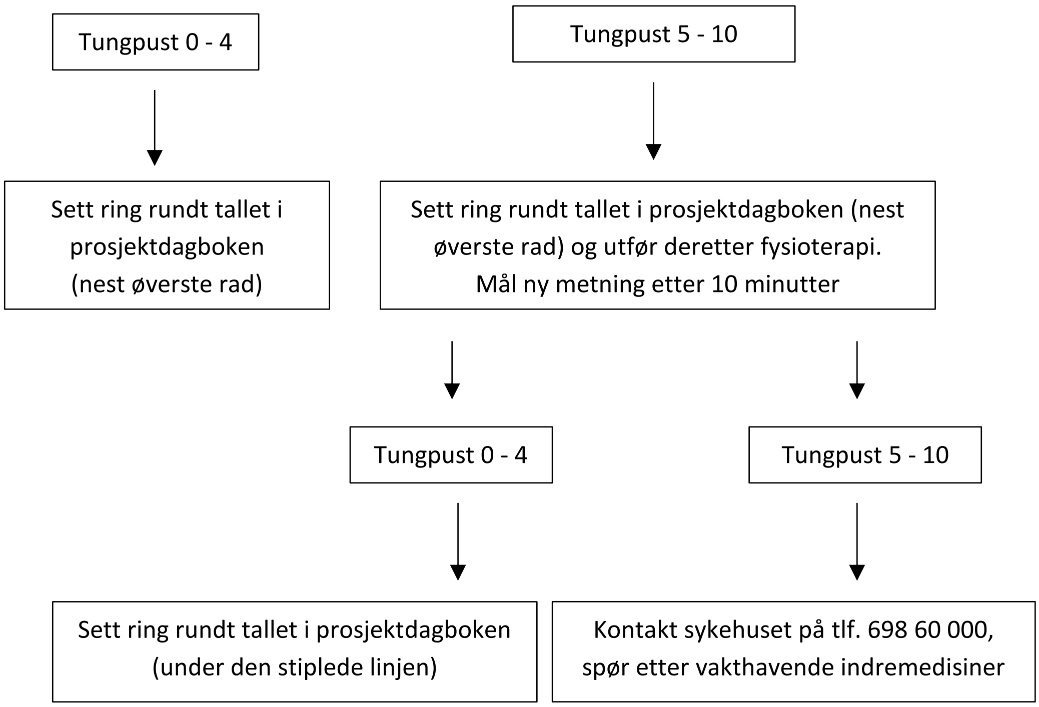

MÅLING AV PUSTEKRAFT

Etter du har målt metningen og angitt graden av tungpust, skal du måle pustekraften (også kalt PEF). Dette gjøres ved å blåse inn i et lite håndholdt apparat (se bildet under). Du begynner med å trekke pusten maksimalt inn, deretter omslutte leppene tett omkring munnstykket på apparatet før du blåser ut så raskt og med så mye kraft du orker. Tallet som vises på apparatet, noterer du i prosjektdagboken.

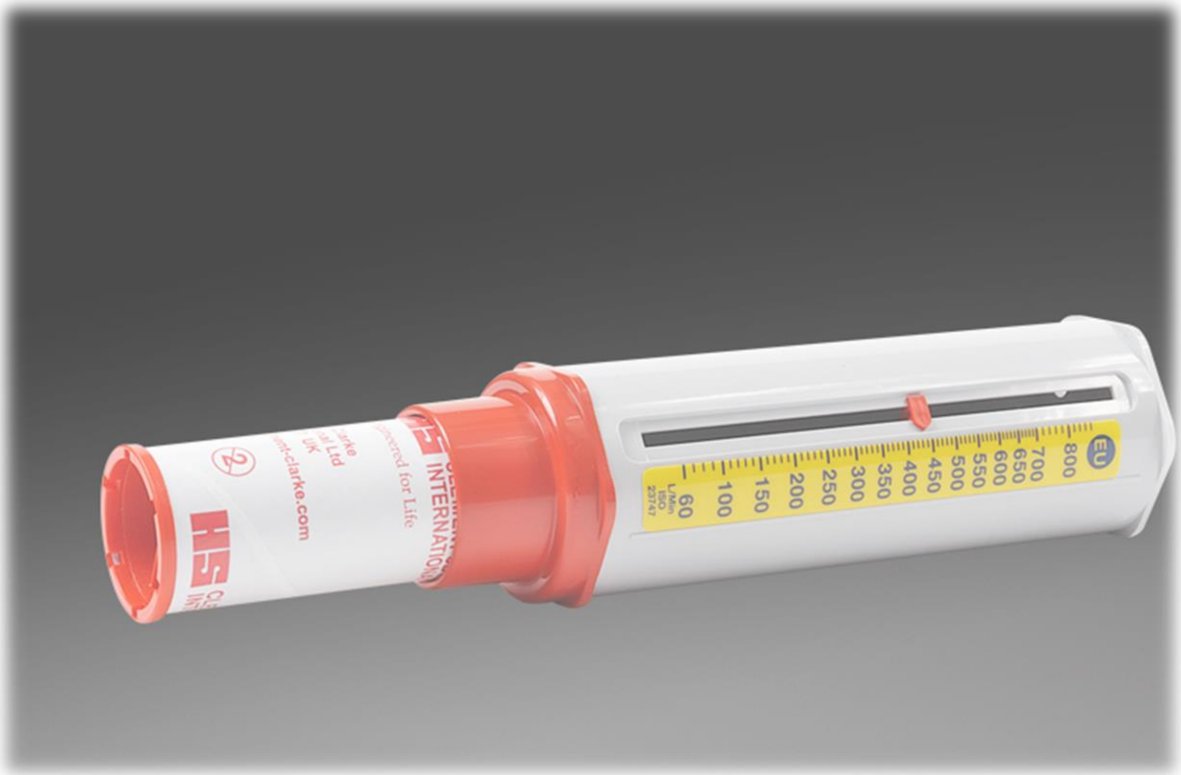

Supplement: Supplementary file 3 — Additional file 3. [file 40814_2023_1415_MOESM3_ESM.pdf]
