# Supplementary material for: The feasibility of self-performing measurements of peripheral oxygen saturation and respiratory exercises in home-isolated COVID-19 patients—a single-arm prospective trial
Source: Pilot Feasibility Stud. 2023 Dec 2;9:195. doi: 10.1186/s40814-023-01415-x (PMC10693052; doi:10.1186/s40814-023-01415-x)

SYKEHUSET ØSTFOLD

SHYCOV - PROSJEKTDAGBOK

Navn

....

Fødselsdag

....

DAG 1

Dato: ....

Kl 08:00

Oksygenmetning (sett ring rundt tallet)

100 99 98 97 96 95 94 93 | 92 ...

Se bakside

Tungpust (sett ring rundt tallet)
0 = ingen tungpust
10 = så tungpusten det går an å bli

0 1 2 3 4 | 5 6 7 8 9 10

Se bakside

Pustekraft (skriv ned tallet)

....

Hvis du har hatt lav metning eller alvorlig tungpust og gjort fysioterapi, fyll ut på nytt under

Oksygenmetning (sett ring rundt tallet)

100 99 98 97 96 95 94 93 | 92 ...

Se bakside

Tungpust (sett ring rundt tallet)
0 = ingen tungpust
10 = så tungpusten det går an å bli

0 1 2 3 4 | 5 6 7 8 9 10

Se bakside

Kl 12:00

Husk fysioterapi-programmet før du går videre

Oksygenmetning (sett ring rundt tallet)

100 99 98 97 96 95 94 93 | 92 ...

Se bakside

Tungpust (sett ring rundt tallet)
0 = ingen tungpust
10 = så tungpusten det går an å bli

0 1 2 3 4 | 5 6 7 8 9 10

Se bakside

Pustekraft (skriv ned tallet)

....

Fikk du utført kl. 12:00 fysioterapi-programmet?

☐ Ja
Antall minutter: ....
☐ Nei

Hvis du har hatt lav metning eller alvorlig tungpust og gjort fysioterapi, fyll ut på nytt under

Oksygenmetning (sett ring rundt tallet)

100 99 98 97 96 95 94 93 | 92 ...

Se bakside

Tungpust (sett ring rundt tallet)
0 = ingen tungpust
10 = så tungpusten det går an å bli

0 1 2 3 4 | 5 6 7 8 9 10

Se bakside

Kl 16:00

Oksygenmetning (sett ring rundt tallet)

100 99 98 97 96 95 94 93 | 92 ...

Se bakside

Tungpust (sett ring rundt tallet)
0 = ingen tungpust
10 = så tungpusten det går an å bli

0 1 2 3 4 | 5 6 7 8 9 10

Se bakside

Pustekraft (skriv ned tallet)

....

Forkjølelsessymptomer (kryss for ja)

- ☐ Feber
  Temp, hvis målt: ....
- ☐ Muskelverk
- ☐ Slapphet
- ☐ Hoste
- ☐ Sår hals
- ☐ Tett nese
- ☐ Kvalme / oppkast
- ☐ Diaré
- ☐ Redusert luktesans

Hvis du har hatt lav metning eller alvorlig tungpust og gjort fysioterapi, fyll ut på nytt under

Oksygenmetning (sett ring rundt tallet)

100 99 98 97 96 95 94 93 | 92 ...

Se bakside

Tungpust (sett ring rundt tallet)
0 = ingen tungpust
10 = så tungpusten det går an å bli

0 1 2 3 4 | 5 6 7 8 9 10

Se bakside

Kl 20:00

Husk fysioterapi-programmet før du går videre

Oksygenmetning (sett ring rundt tallet)

100 99 98 97 96 95 94 93 | 92 ...

Se bakside

Tungpust (sett ring rundt tallet)
0 = ingen tungpust
10 = så tungpusten det går an å bli

0 1 2 3 4 | 5 6 7 8 9 10

Se bakside

Pustekraft (skriv ned tallet)

....

Aktivitetsnivå i løpet av dagen

☐ Normalt ☐ Litt redusert ☐ Veldig redusert

Fikk du utført kl. 20:00 fysioterapi-programmet?

- ☐ Ja
  Antall minutter: ....
- ☐ Nei

Hvis du har hatt lav metning eller alvorlig tungpust og gjort fysioterapi, fyll ut på nytt under

Oksygenmetning (sett ring rundt tallet)

100 99 98 97 96 95 94 93 | 92 ...

Se bakside

Tungpust (sett ring rundt tallet)
0 = ingen tungpust
10 = så tungpusten det går an å bli

0 1 2 3 4 | 5 6 7 8 9 10

Se bakside

DAG 2

Dato: ....

Kl 08:00

Oksygenmetning (sett ring rundt tallet)

100 99 98 97 96 95 94 93 | 92 ...

Se bakside

Tungpust (sett ring rundt tallet)
0 = ingen tungpust
10 = så tungpusten det går an å bli

0 1 2 3 4 | 5 6 7 8 9 10

Se bakside

Pustekraft (skriv ned tallet)

....

Hvis du har hatt lav metning eller alvorlig tungpust og gjort fysioterapi, fyll ut på nytt under

Oksygenmetning (sett ring rundt tallet)

100 99 98 97 96 95 94 93 | 92 ...

Se bakside

Tungpust (sett ring rundt tallet)
0 = ingen tungpust
10 = så tungpusten det går an å bli

0 1 2 3 4 | 5 6 7 8 9 10

Se bakside

Kl 12:00

Husk fysioterapi-programmet før du går videre

Oksygenmetning (sett ring rundt tallet)

100 99 98 97 96 95 94 93 | 92 ...

Se bakside

Tungpust (sett ring rundt tallet)
0 = ingen tungpust
10 = så tungpusten det går an å bli

0 1 2 3 4 | 5 6 7 8 9 10

Se bakside

Pustekraft (skriv ned tallet)

....

Fikk du utført kl. 12:00 fysioterapi-programmet?

☐ Ja

Antall minutter: ....

☐ Nei

Hvis du har hatt lav metning eller alvorlig tungpust og gjort fysioterapi, fyll ut på nytt under

Oksygenmetning (sett ring rundt tallet)

100 99 98 97 96 95 94 93 | 92 ...

Se bakside

Tungpust (sett ring rundt tallet)
0 = ingen tungpust
10 = så tungpusten det går an å bli

0 1 2 3 4 | 5 6 7 8 9 10

Se bakside

Kl 16:00

Oksygenmetning (sett ring rundt tallet)

100 99 98 97 96 95 94 93 | 92 ...

Se bakside

Tungpust (sett ring rundt tallet)
0 = ingen tungpust
10 = så tungpusten det går an å bli

0 1 2 3 4 | 5 6 7 8 9 10

Se bakside

Pustekraft (skriv ned tallet)

....

Forkjølelsessymptomer (kryss for ja)

- ☐ Feber
  - Temp, hvis målt: ....
- ☐ Muskelverk
- ☐ Slapphet
- ☐ Hoste
- ☐ Sår hals
- ☐ Tett nese
- ☐ Kvalme / oppkast
- ☐ Diaré
- ☐ Redusert luktesans

Hvis du har hatt lav metning eller alvorlig tungpust og gjort fysioterapi, fyll ut på nytt under

Oksygenmetning (sett ring rundt tallet)

100 99 98 97 96 95 94 93 | 92 ...

Se bakside

Tungpust (sett ring rundt tallet)
0 = ingen tungpust
10 = så tungpusten det går an å bli

0 1 2 3 4 | 5 6 7 8 9 10

Se bakside

Kl 20:00

Husk fysioterapi-programmet før du går videre

Oksygenmetning (sett ring rundt tallet)

100 99 98 97 96 95 94 93 | 92 ...

Se bakside

Tungpust (sett ring rundt tallet)
0 = ingen tungpust
10 = så tungpusten det går an å bli

0 1 2 3 4 | 5 6 7 8 9 10

Se bakside

Pustekraft (skriv ned tallet)

....

Aktivitetsnivå i løpet av dagen

☐ Normalt ☐ Litt redusert ☐ Veldig redusert

Fikk du utført kl. 20:00 fysioterapi-programmet?

- ☐ Ja
  - Antall minutter: ....
- ☐ Nei

Hvis du har hatt lav metning eller alvorlig tungpust og gjort fysioterapi, fyll ut på nytt under

Oksygenmetning (sett ring rundt tallet)

100 99 98 97 96 95 94 93 | 92 ...

Se bakside

Tungpust (sett ring rundt tallet)
0 = ingen tungpust
10 = så tungpusten det går an å bli

0 1 2 3 4 | 5 6 7 8 9 10

Se bakside

DAG 3

Dato: ....

Kl 08:00

Oksygenmetning (sett ring rundt tallet)

100 99 98 97 96 95 94 93 | 92 ...

Se bakside

Tungpust (sett ring rundt tallet)
0 = ingen tungpust
10 = så tungpusten det går an å bli

0 1 2 3 4 | 5 6 7 8 9 10

Se bakside

Pustekraft (skriv ned tallet)

....

Hvis du har hatt lav metning eller alvorlig tungpust og gjort fysioterapi, fyll ut på nytt under

Oksygenmetning (sett ring rundt tallet)

100 99 98 97 96 95 94 93 | 92 ...

Se bakside

Tungpust (sett ring rundt tallet)
0 = ingen tungpust
10 = så tungpusten det går an å bli

0 1 2 3 4 | 5 6 7 8 9 10

Se bakside

Kl 12:00

Husk fysioterapi-programmet før du går videre

Oksygenmetning (sett ring rundt tallet)

100 99 98 97 96 95 94 93 | 92 ...

Se bakside

Tungpust (sett ring rundt tallet)
0 = ingen tungpust
10 = så tungpusten det går an å bli

0 1 2 3 4 | 5 6 7 8 9 10

Se bakside

Pustekraft (skriv ned tallet)

....

Fikk du utført kl. 12:00 fysioterapi-programmet?

☐ Ja
Antall minutter: ....
☐ Nei

Hvis du har hatt lav metning eller alvorlig tungpust og gjort fysioterapi, fyll ut på nytt under

Oksygenmetning (sett ring rundt tallet)

100 99 98 97 96 95 94 93 | 92 ...

Se bakside

Tungpust (sett ring rundt tallet)
0 = ingen tungpust
10 = så tungpusten det går an å bli

0 1 2 3 4 | 5 6 7 8 9 10

Se bakside

Kl 16:00

Oksygenmetning (sett ring rundt tallet)

100 99 98 97 96 95 94 93 | 92 ...

Se bakside

Tungpust (sett ring rundt tallet)
0 = ingen tungpust
10 = så tungpusten det går an å bli

0 1 2 3 4 | 5 6 7 8 9 10

Se bakside

Pustekraft (skriv ned tallet)

....

Forkjølelsessymptomer (kryss for ja)

- ☐ Feber
  Temp, hvis målt: ....
- ☐ Muskelverk
- ☐ Slapphet
- ☐ Hoste
- ☐ Sår hals
- ☐ Tett nese
- ☐ Kvalme / oppkast
- ☐ Diaré
- ☐ Redusert luktesans

Hvis du har hatt lav metning eller alvorlig tungpust og gjort fysioterapi, fyll ut på nytt under

Oksygenmetning (sett ring rundt tallet)

100 99 98 97 96 95 94 93 | 92 ...

Se bakside

Tungpust (sett ring rundt tallet)
0 = ingen tungpust
10 = så tungpusten det går an å bli

0 1 2 3 4 | 5 6 7 8 9 10

Se bakside

Kl 20:00

Husk fysioterapi-programmet før du går videre

Oksygenmetning (sett ring rundt tallet)

100 99 98 97 96 95 94 93 | 92 ...

Se bakside

Tungpust (sett ring rundt tallet)
0 = ingen tungpust
10 = så tungpusten det går an å bli

0 1 2 3 4 | 5 6 7 8 9 10

Se bakside

Pustekraft (skriv ned tallet)

....

Aktivitetsnivå i løpet av dagen

☐ Normalt ☐ Litt redusert ☐ Veldig redusert

Fikk du utført kl. 20:00 fysioterapi-programmet?

- ☐ Ja
  Antall minutter: ....
- ☐ Nei

Hvis du har hatt lav metning eller alvorlig tungpust og gjort fysioterapi, fyll ut på nytt under

Oksygenmetning (sett ring rundt tallet)

100 99 98 97 96 95 94 93 | 92 ...

Se bakside

Tungpust (sett ring rundt tallet)
0 = ingen tungpust
10 = så tungpusten det går an å bli

0 1 2 3 4 | 5 6 7 8 9 10

Se bakside

DAG 4

Dato: ....

Kl 08:00

Oksygenmetning (sett ring rundt tallet)

100 99 98 97 96 95 94 93 | 92 ...

Se bakside

Tungpust (sett ring rundt tallet)
0 = ingen tungpust
10 = så tungpusten det går an å bli

0 1 2 3 4 | 5 6 7 8 9 10

Se bakside

Pustekraft (skriv ned tallet)

....

Hvis du har hatt lav metning eller alvorlig tungpust og gjort fysioterapi, fyll ut på nytt under

Oksygenmetning (sett ring rundt tallet)

100 99 98 97 96 95 94 93 | 92 ...

Se bakside

Tungpust (sett ring rundt tallet)
0 = ingen tungpust
10 = så tungpusten det går an å bli

0 1 2 3 4 | 5 6 7 8 9 10

Se bakside

Kl 12:00

Husk fysioterapi-programmet før du går videre

Oksygenmetning (sett ring rundt tallet)

100 99 98 97 96 95 94 93 | 92 ...

Se bakside

Tungpust (sett ring rundt tallet)
0 = ingen tungpust
10 = så tungpusten det går an å bli

0 1 2 3 4 | 5 6 7 8 9 10

Se bakside

Pustekraft (skriv ned tallet)

....

Fikk du utført kl. 12:00 fysioterapi-programmet?

☐ Ja
Antall minutter: ....
☐ Nei

Hvis du har hatt lav metning eller alvorlig tungpust og gjort fysioterapi, fyll ut på nytt under

Oksygenmetning (sett ring rundt tallet)

100 99 98 97 96 95 94 93 | 92 ...

Se bakside

Tungpust (sett ring rundt tallet)
0 = ingen tungpust
10 = så tungpusten det går an å bli

0 1 2 3 4 | 5 6 7 8 9 10

Se bakside

Kl 16:00

Oksygenmetning (sett ring rundt tallet)

100 99 98 97 96 95 94 93 92 ...

Se bakside

Tungpust (sett ring rundt tallet)
0 = ingen tungpust
10 = så tungpusten det går an å bli

0 1 2 3 4 5 6 7 8 9 10

Se bakside

Pustekraft (skriv ned tallet)

....

Forkjølelsessymptomer (kryss for ja)

- ☐ Feber
  Temp, hvis målt: ....
- ☐ Muskelverk
- ☐ Slapphet
- ☐ Hoste
- ☐ Sår hals
- ☐ Tett nese
- ☐ Kvalme / oppkast
- ☐ Diaré
- ☐ Redusert luktesans

Hvis du har hatt lav metning eller alvorlig tungpust og gjort fysioterapi, fyll ut på nytt under

Oksygenmetning (sett ring rundt tallet)

100 99 98 97 96 95 94 93 92 ...

Se bakside

Tungpust (sett ring rundt tallet)
0 = ingen tungpust
10 = så tungpusten det går an å bli

0 1 2 3 4 5 6 7 8 9 10

Se bakside

Kl 20:00

Husk fysioterapi-programmet før du går videre

Oksygenmetning (sett ring rundt tallet)

100 99 98 97 96 95 94 93 92 ...

Se bakside

Tungpust (sett ring rundt tallet)
0 = ingen tungpust
10 = så tungpusten det går an å bli

0 1 2 3 4 5 6 7 8 9 10

Se bakside

Pustekraft (skriv ned tallet)

....

Aktivitetsnivå i løpet av dagen

☐ Normalt ☐ Litt redusert ☐ Veldig redusert

Fikk du utført kl. 20:00 fysioterapi-programmet?

- ☐ Ja
  Antall minutter: ....
- ☐ Nei

Hvis du har hatt lav metning eller alvorlig tungpust og gjort fysioterapi, fyll ut på nytt under

Oksygenmetning (sett ring rundt tallet)

100 99 98 97 96 95 94 93 92 ...

Se bakside

Tungpust (sett ring rundt tallet)
0 = ingen tungpust
10 = så tungpusten det går an å bli

0 1 2 3 4 5 6 7 8 9 10

Se bakside

DAG 5

Dato: ....

Kl 08:00

Oksygenmetning (sett ring rundt tallet)

100 99 98 97 96 95 94 93 | 92 ...

Se bakside

Tungpust (sett ring rundt tallet)
0 = ingen tungpust
10 = så tungpusten det går an å bli

0 1 2 3 4 | 5 6 7 8 9 10

Se bakside

Pustekraft (skriv ned tallet)

....

Hvis du har hatt lav metning eller alvorlig tungpust og gjort fysioterapi, fyll ut på nytt under

Oksygenmetning (sett ring rundt tallet)

100 99 98 97 96 95 94 93 | 92 ...

Se bakside

Tungpust (sett ring rundt tallet)
0 = ingen tungpust
10 = så tungpusten det går an å bli

0 1 2 3 4 | 5 6 7 8 9 10

Se bakside

Kl 12:00

Husk fysioterapi-programmet før du går videre

Oksygenmetning (sett ring rundt tallet)

100 99 98 97 96 95 94 93 | 92 ...

Se bakside

Tungpust (sett ring rundt tallet)
0 = ingen tungpust
10 = så tungpusten det går an å bli

0 1 2 3 4 | 5 6 7 8 9 10

Se bakside

Pustekraft (skriv ned tallet)

....

Fikk du utført kl. 12:00 fysioterapi-programmet?

☐ Ja
Antall minutter: ....
☐ Nei

Hvis du har hatt lav metning eller alvorlig tungpust og gjort fysioterapi, fyll ut på nytt under

Oksygenmetning (sett ring rundt tallet)

100 99 98 97 96 95 94 93 | 92 ...

Se bakside

Tungpust (sett ring rundt tallet)
0 = ingen tungpust
10 = så tungpusten det går an å bli

0 1 2 3 4 | 5 6 7 8 9 10

Se bakside

Kl 16:00

Oksygenmetning (sett ring rundt tallet)

100 99 98 97 96 95 94 93 92 ...

Se bakside

Tungpust (sett ring rundt tallet)

0 = ingen tungpust
10 = så tungpusten det går an å bli

0 1 2 3 4 5 6 7 8 9 10

Se bakside

Pustekraft (skriv ned tallet)

....

Forkjølelsessymptomer (kryss for ja)

- ☐ Feber
  Temp, hvis målt: ....
- ☐ Muskelverk
- ☐ Slapphet
- ☐ Hoste
- ☐ Sår hals
- ☐ Tett nese
- ☐ Kvalme / oppkast
- ☐ Diaré
- ☐ Redusert luktesans

Hvis du har hatt lav metning eller alvorlig tungpust og gjort fysioterapi, fyll ut på nytt under

Oksygenmetning (sett ring rundt tallet)

100 99 98 97 96 95 94 93 92 ...

Se bakside

Tungpust (sett ring rundt tallet)

0 = ingen tungpust
10 = så tungpusten det går an å bli

0 1 2 3 4 5 6 7 8 9 10

Se bakside

Kl 20:00

Husk fysioterapi-programmet før du går videre

Oksygenmetning (sett ring rundt tallet)

100 99 98 97 96 95 94 93 92 ...

Se bakside

Tungpust (sett ring rundt tallet)

0 = ingen tungpust
10 = så tungpusten det går an å bli

0 1 2 3 4 5 6 7 8 9 10

Se bakside

Pustekraft (skriv ned tallet)

....

Aktivitetsnivå i løpet av dagen

☐ Normalt ☐ Litt redusert ☐ Veldig redusert

Fikk du utført kl. 20:00 fysioterapi-programmet?

- ☐ Ja
  Antall minutter: ....
- ☐ Nei

Hvis du har hatt lav metning eller alvorlig tungpust og gjort fysioterapi, fyll ut på nytt under

Oksygenmetning (sett ring rundt tallet)

100 99 98 97 96 95 94 93 92 ...

Se bakside

Tungpust (sett ring rundt tallet)

0 = ingen tungpust
10 = så tungpusten det går an å bli

0 1 2 3 4 5 6 7 8 9 10

Se bakside

DAG 6

Dato: ....

Kl 08:00

Oksygenmetning (sett ring rundt tallet)

100 99 98 97 96 95 94 93 92 ...

Se bakside

Tungpust (sett ring rundt tallet)
0 = ingen tungpust
10 = så tungpusten det går an å bli

0 1 2 3 4 5 6 7 8 9 10

Se bakside

Pustekraft (skriv ned tallet)

....

Hvis du har hatt lav metning eller alvorlig tungpust og gjort fysioterapi, fyll ut på nytt under

Oksygenmetning (sett ring rundt tallet)

100 99 98 97 96 95 94 93 92 ...

Se bakside

Tungpust (sett ring rundt tallet)
0 = ingen tungpust
10 = så tungpusten det går an å bli

0 1 2 3 4 5 6 7 8 9 10

Se bakside

Kl 12:00

Husk fysioterapi-programmet før du går videre

Oksygenmetning (sett ring rundt tallet)

100 99 98 97 96 95 94 93 92 ...

Se bakside

Tungpust (sett ring rundt tallet)
0 = ingen tungpust
10 = så tungpusten det går an å bli

0 1 2 3 4 5 6 7 8 9 10

Se bakside

Pustekraft (skriv ned tallet)

....

Fikk du utført kl. 12:00 fysioterapi-programmet?

☐ Ja
Antall minutter: ....
☐ Nei

Hvis du har hatt lav metning eller alvorlig tungpust og gjort fysioterapi, fyll ut på nytt under

Oksygenmetning (sett ring rundt tallet)

100 99 98 97 96 95 94 93 92 ...

Se bakside

Tungpust (sett ring rundt tallet)
0 = ingen tungpust
10 = så tungpusten det går an å bli

0 1 2 3 4 5 6 7 8 9 10

Se bakside

Kl 16:00

Oksygenmetning (sett ring rundt tallet)

100 99 98 97 96 95 94 93 92 ...

Se bakside

Tungpust (sett ring rundt tallet)
0 = ingen tungpust
10 = så tungpusten det går an å bli

0 1 2 3 4 5 6 7 8 9 10

Se bakside

Pustekraft (skriv ned tallet)

....

Forkjølelsessymptomer (kryss for ja)

- ☐ Feber
  Temp, hvis målt: ....
- ☐ Muskelverk
- ☐ Slapphet
- ☐ Hoste
- ☐ Sår hals
- ☐ Tett nese
- ☐ Kvalme / oppkast
- ☐ Diaré
- ☐ Redusert luktesans

Hvis du har hatt lav metning eller alvorlig tungpust og gjort fysioterapi, fyll ut på nytt under

Oksygenmetning (sett ring rundt tallet)

100 99 98 97 96 95 94 93 92 ...

Se bakside

Tungpust (sett ring rundt tallet)
0 = ingen tungpust
10 = så tungpusten det går an å bli

0 1 2 3 4 5 6 7 8 9 10

Se bakside

Kl 20:00

Husk fysioterapi-programmet før du går videre

Oksygenmetning (sett ring rundt tallet)

100 99 98 97 96 95 94 93 92 ...

Se bakside

Tungpust (sett ring rundt tallet)
0 = ingen tungpust
10 = så tungpusten det går an å bli

0 1 2 3 4 5 6 7 8 9 10

Se bakside

Pustekraft (skriv ned tallet)

....

Aktivitetsnivå i løpet av dagen

☐ Normalt ☐ Litt redusert ☐ Veldig redusert

Fikk du utført kl. 20:00 fysioterapi-programmet?

- ☐ Ja
  Antall minutter: ....
- ☐ Nei

Hvis du har hatt lav metning eller alvorlig tungpust og gjort fysioterapi, fyll ut på nytt under

Oksygenmetning (sett ring rundt tallet)

100 99 98 97 96 95 94 93 92 ...

Se bakside

Tungpust (sett ring rundt tallet)
0 = ingen tungpust
10 = så tungpusten det går an å bli

0 1 2 3 4 5 6 7 8 9 10

Se bakside

DAG 7

Dato: ....

Kl 08:00

Oksygenmetning (sett ring rundt tallet)

100 99 98 97 96 95 94 93 | 92 ...

Se bakside

Tungpust (sett ring rundt tallet)
0 = ingen tungpust
10 = så tungpusten det går an å bli

0 1 2 3 4 | 5 6 7 8 9 10

Se bakside

Pustekraft (skriv ned tallet)

....

Kl 12:00

Husk fysioterapi-programmet før du går videre

Oksygenmetning (sett ring rundt tallet)

100 99 98 97 96 95 94 93 | 92 ...

Se bakside

Tungpust (sett ring rundt tallet)
0 = ingen tungpust
10 = så tungpusten det går an å bli

0 1 2 3 4 | 5 6 7 8 9 10

Se bakside

Pustekraft (skriv ned tallet)

....

Fikk du utført kl. 12:00 fysioterapi-programmet?

☐ Ja
Antall minutter: ....
☐ Nei

Hvis du har hatt lav metning eller alvorlig tungpust og gjort fysioterapi, fyll ut på nytt under

Oksygenmetning (sett ring rundt tallet)

100 99 98 97 96 95 94 93 | 92 ...

Se bakside

Tungpust (sett ring rundt tallet)
0 = ingen tungpust
10 = så tungpusten det går an å bli

0 1 2 3 4 | 5 6 7 8 9 10

Se bakside

Hvis du har hatt lav metning eller alvorlig tungpust og gjort fysioterapi, fyll ut på nytt under

Oksygenmetning (sett ring rundt tallet)

100 99 98 97 96 95 94 93 | 92 ...

Se bakside

Tungpust (sett ring rundt tallet)
0 = ingen tungpust
10 = så tungpusten det går an å bli

0 1 2 3 4 | 5 6 7 8 9 10

Se bakside

Kl 16:00

Oksygenmetning (sett ring rundt tallet)

100 99 98 97 96 95 94 93 | 92 ...

Se bakside

Tungpust (sett ring rundt tallet)
0 = ingen tungpust
10 = så tungpusten det går an å bli

0 1 2 3 4 | 5 6 7 8 9 10

Se bakside

Pustekraft (skriv ned tallet)

....

Forkjølelsessymptomer (kryss for ja)

- ☐ Feber
  Temp, hvis målt: ....
- ☐ Muskelverk
- ☐ Slapphet
- ☐ Hoste
- ☐ Sår hals
- ☐ Tett nese
- ☐ Kvalme / oppkast
- ☐ Diaré
- ☐ Redusert luktesans

Hvis du har hatt lav metning eller alvorlig tungpust og gjort fysioterapi, fyll ut på nytt under

Oksygenmetning (sett ring rundt tallet)

100 99 98 97 96 95 94 93 | 92 ...

Se bakside

Tungpust (sett ring rundt tallet)
0 = ingen tungpust
10 = så tungpusten det går an å bli

0 1 2 3 4 | 5 6 7 8 9 10

Se bakside

Kl 20:00

Husk fysioterapi-programmet før du går videre

Oksygenmetning (sett ring rundt tallet)

100 99 98 97 96 95 94 93 | 92 ...

Se bakside

Tungpust (sett ring rundt tallet)
0 = ingen tungpust
10 = så tungpusten det går an å bli

0 1 2 3 4 | 5 6 7 8 9 10

Se bakside

Pustekraft (skriv ned tallet)

....

Aktivitetsnivå i løpet av dagen

☐ Normalt ☐ Litt redusert ☐ Veldig redusert

Fikk du utført kl. 20:00 fysioterapi-programmet?

- ☐ Ja
  Antall minutter: ....
- ☐ Nei

Hvis du har hatt lav metning eller alvorlig tungpust og gjort fysioterapi, fyll ut på nytt under

Oksygenmetning (sett ring rundt tallet)

100 99 98 97 96 95 94 93 | 92 ...

Se bakside

Tungpust (sett ring rundt tallet)
0 = ingen tungpust
10 = så tungpusten det går an å bli

0 1 2 3 4 | 5 6 7 8 9 10

Se bakside

DAG 8

Dato: ....

Kl 08:00

Oksygenmetning (sett ring rundt tallet)

100 99 98 97 96 95 94 93 | 92 ...

Se bakside

Tungpust (sett ring rundt tallet)
0 = ingen tungpust
10 = så tungpusten det går an å bli

0 1 2 3 4 | 5 6 7 8 9 10

Se bakside

Pustekraft (skriv ned tallet)

....

Kl 12:00

Husk fysioterapi-programmet før du går videre

Oksygenmetning (sett ring rundt tallet)

100 99 98 97 96 95 94 93 | 92 ...

Se bakside

Tungpust (sett ring rundt tallet)
0 = ingen tungpust
10 = så tungpusten det går an å bli

0 1 2 3 4 | 5 6 7 8 9 10

Se bakside

Pustekraft (skriv ned tallet)

....

Fikk du utført kl. 12:00 fysioterapi-programmet?

☐ Ja
Antall minutter: ....
☐ Nei

Hvis du har hatt lav metning eller alvorlig tungpust og gjort fysioterapi, fyll ut på nytt under

Oksygenmetning (sett ring rundt tallet)

100 99 98 97 96 95 94 93 | 92 ...

Se bakside

Tungpust (sett ring rundt tallet)
0 = ingen tungpust
10 = så tungpusten det går an å bli

0 1 2 3 4 | 5 6 7 8 9 10

Se bakside

Hvis du har hatt lav metning eller alvorlig tungpust og gjort fysioterapi, fyll ut på nytt under

Oksygenmetning (sett ring rundt tallet)

100 99 98 97 96 95 94 93 | 92 ...

Se bakside

Tungpust (sett ring rundt tallet)
0 = ingen tungpust
10 = så tungpusten det går an å bli

0 1 2 3 4 | 5 6 7 8 9 10

Se bakside

Kl 16:00

Oksygenmetning (sett ring rundt tallet)

100 99 98 97 96 95 94 93 | 92 ...

Se bakside

Tungpust (sett ring rundt tallet)
0 = ingen tungpust
10 = så tungpusten det går an å bli

0 1 2 3 4 | 5 6 7 8 9 10

Se bakside

Pustekraft (skriv ned tallet)

....

Forkjølelsessymptomer (kryss for ja)

- ☐ Feber
  - Temp, hvis målt: ....
- ☐ Muskelverk
- ☐ Slapphet
- ☐ Hoste
- ☐ Sår hals
- ☐ Tett nese
- ☐ Kvalme / oppkast
- ☐ Diaré
- ☐ Redusert luktesans

Hvis du har hatt lav metning eller alvorlig tungpust og gjort fysioterapi, fyll ut på nytt under

Oksygenmetning (sett ring rundt tallet)

100 99 98 97 96 95 94 93 | 92 ...

Se bakside

Tungpust (sett ring rundt tallet)
0 = ingen tungpust
10 = så tungpusten det går an å bli

0 1 2 3 4 | 5 6 7 8 9 10

Se bakside

Kl 20:00

Husk fysioterapi-programmet før du går videre

Oksygenmetning (sett ring rundt tallet)

100 99 98 97 96 95 94 93 | 92 ...

Se bakside

Tungpust (sett ring rundt tallet)
0 = ingen tungpust
10 = så tungpusten det går an å bli

0 1 2 3 4 | 5 6 7 8 9 10

Se bakside

Pustekraft (skriv ned tallet)

....

Aktivitetsnivå i løpet av dagen

☐ Normalt ☐ Litt redusert ☐ Veldig redusert

Fikk du utført kl. 20:00 fysioterapi-programmet?

- ☐ Ja
  - Antall minutter: ....
- ☐ Nei

Hvis du har hatt lav metning eller alvorlig tungpust og gjort fysioterapi, fyll ut på nytt under

Oksygenmetning (sett ring rundt tallet)

100 99 98 97 96 95 94 93 | 92 ...

Se bakside

Tungpust (sett ring rundt tallet)
0 = ingen tungpust
10 = så tungpusten det går an å bli

0 1 2 3 4 | 5 6 7 8 9 10

Se bakside

DAG 9

Dato: ....

Kl 08:00

Oksygenmetning (sett ring rundt tallet)

100 99 98 97 96 95 94 93 | 92 ...

Se bakside

Tungpust (sett ring rundt tallet)
0 = ingen tungpust
10 = så tungpusten det går an å bli

0 1 2 3 4 | 5 6 7 8 9 10

Se bakside

Pustekraft (skriv ned tallet)

....

Hvis du har hatt lav metning eller alvorlig tungpust og gjort fysioterapi, fyll ut på nytt under

Oksygenmetning (sett ring rundt tallet)

100 99 98 97 96 95 94 93 | 92 ...

Se bakside

Tungpust (sett ring rundt tallet)
0 = ingen tungpust
10 = så tungpusten det går an å bli

0 1 2 3 4 | 5 6 7 8 9 10

Se bakside

Kl 12:00

Husk fysioterapi-programmet før du går videre

Oksygenmetning (sett ring rundt tallet)

100 99 98 97 96 95 94 93 | 92 ...

Se bakside

Tungpust (sett ring rundt tallet)
0 = ingen tungpust
10 = så tungpusten det går an å bli

0 1 2 3 4 | 5 6 7 8 9 10

Se bakside

Pustekraft (skriv ned tallet)

....

Fikk du utført kl. 12:00 fysioterapi-programmet?

☐ Ja

Antall minutter: ....

☐ Nei

Hvis du har hatt lav metning eller alvorlig tungpust og gjort fysioterapi, fyll ut på nytt under

Oksygenmetning (sett ring rundt tallet)

100 99 98 97 96 95 94 93 | 92 ...

Se bakside

Tungpust (sett ring rundt tallet)
0 = ingen tungpust
10 = så tungpusten det går an å bli

0 1 2 3 4 | 5 6 7 8 9 10

Se bakside

Kl 16:00

Oksygenmetning (sett ring rundt tallet)

100 99 98 97 96 95 94 93 | 92 ...

Se bakside

Tungpust (sett ring rundt tallet)
0 = ingen tungpust
10 = så tungpusten det går an å bli

0 1 2 3 4 | 5 6 7 8 9 10

Se bakside

Pustekraft (skriv ned tallet)

....

Forkjølelsessymptomer (kryss for ja)

- ☐ Feber
  Temp, hvis målt: ....
- ☐ Muskelverk
- ☐ Slapphet
- ☐ Hoste
- ☐ Sår hals
- ☐ Tett nese
- ☐ Kvalme / oppkast
- ☐ Diaré
- ☐ Redusert luktesans

Hvis du har hatt lav metning eller alvorlig tungpust og gjort fysioterapi, fyll ut på nytt under

Oksygenmetning (sett ring rundt tallet)

100 99 98 97 96 95 94 93 | 92 ...

Se bakside

Tungpust (sett ring rundt tallet)
0 = ingen tungpust
10 = så tungpusten det går an å bli

0 1 2 3 4 | 5 6 7 8 9 10

Se bakside

Kl 20:00

Husk fysioterapi-programmet før du går videre

Oksygenmetning (sett ring rundt tallet)

100 99 98 97 96 95 94 93 | 92 ...

Se bakside

Tungpust (sett ring rundt tallet)
0 = ingen tungpust
10 = så tungpusten det går an å bli

0 1 2 3 4 | 5 6 7 8 9 10

Se bakside

Pustekraft (skriv ned tallet)

....

Aktivitetsnivå i løpet av dagen

☐ Normalt ☐ Litt redusert ☐ Veldig redusert

Fikk du utført kl. 20:00 fysioterapi-programmet?

- ☐ Ja
  Antall minutter: ....
- ☐ Nei

Hvis du har hatt lav metning eller alvorlig tungpust og gjort fysioterapi, fyll ut på nytt under

Oksygenmetning (sett ring rundt tallet)

100 99 98 97 96 95 94 93 | 92 ...

Se bakside

Tungpust (sett ring rundt tallet)
0 = ingen tungpust
10 = så tungpusten det går an å bli

0 1 2 3 4 | 5 6 7 8 9 10

Se bakside

DAG 10

Dato: ....

Kl 08:00

Oksygenmetning (sett ring rundt tallet)

100 99 98 97 96 95 94 93 | 92 ...

Se bakside

Tungpust (sett ring rundt tallet)
0 = ingen tungpust
10 = så tungpusten det går an å bli

0 1 2 3 4 | 5 6 7 8 9 10

Se bakside

Pustekraft (skriv ned tallet)

....

Hvis du har hatt lav metning eller alvorlig tungpust og gjort fysioterapi, fyll ut på nytt under

Oksygenmetning (sett ring rundt tallet)

100 99 98 97 96 95 94 93 | 92 ...

Se bakside

Tungpust (sett ring rundt tallet)
0 = ingen tungpust
10 = så tungpusten det går an å bli

0 1 2 3 4 | 5 6 7 8 9 10

Se bakside

Kl 12:00

Husk fysioterapi-programmet før du går videre

Oksygenmetning (sett ring rundt tallet)

100 99 98 97 96 95 94 93 | 92 ...

Se bakside

Tungpust (sett ring rundt tallet)
0 = ingen tungpust
10 = så tungpusten det går an å bli

0 1 2 3 4 | 5 6 7 8 9 10

Se bakside

Pustekraft (skriv ned tallet)

....

Fikk du utført kl. 12:00 fysioterapi-programmet?

☐ Ja
Antall minutter: ....
☐ Nei

Hvis du har hatt lav metning eller alvorlig tungpust og gjort fysioterapi, fyll ut på nytt under

Oksygenmetning (sett ring rundt tallet)

100 99 98 97 96 95 94 93 | 92 ...

Se bakside

Tungpust (sett ring rundt tallet)
0 = ingen tungpust
10 = så tungpusten det går an å bli

0 1 2 3 4 | 5 6 7 8 9 10

Se bakside

Kl 16:00

Oksygenmetning (sett ring rundt tallet)

100 99 98 97 96 95 94 93 | 92 ...

Se bakside

Tungpust (sett ring rundt tallet)
0 = ingen tungpust
10 = så tungpusten det går an å bli

0 1 2 3 4 | 5 6 7 8 9 10

Se bakside

Pustekraft (skriv ned tallet)

....

Forkjølelsessymptomer (kryss for ja)

- ☐ Feber
  Temp, hvis målt: ....
- ☐ Muskelverk
- ☐ Slapphet
- ☐ Hoste
- ☐ Sår hals
- ☐ Tett nese
- ☐ Kvalme / oppkast
- ☐ Diaré
- ☐ Redusert luktesans

Hvis du har hatt lav metning eller alvorlig tungpust og gjort fysioterapi, fyll ut på nytt under

Oksygenmetning (sett ring rundt tallet)

100 99 98 97 96 95 94 93 | 92 ...

Se bakside

Tungpust (sett ring rundt tallet)
0 = ingen tungpust
10 = så tungpusten det går an å bli

0 1 2 3 4 | 5 6 7 8 9 10

Se bakside

Kl 20:00

Husk fysioterapi-programmet før du går videre

Oksygenmetning (sett ring rundt tallet)

100 99 98 97 96 95 94 93 | 92 ...

Se bakside

Tungpust (sett ring rundt tallet)
0 = ingen tungpust
10 = så tungpusten det går an å bli

0 1 2 3 4 | 5 6 7 8 9 10

Se bakside

Pustekraft (skriv ned tallet)

....

Aktivitetsnivå i løpet av dagen

☐ Normalt ☐ Litt redusert ☐ Veldig redusert

Fikk du utført kl. 20:00 fysioterapi-programmet?

- ☐ Ja
  Antall minutter: ....
- ☐ Nei

Hvis du har hatt lav metning eller alvorlig tungpust og gjort fysioterapi, fyll ut på nytt under

Oksygenmetning (sett ring rundt tallet)

100 99 98 97 96 95 94 93 | 92 ...

Se bakside

Tungpust (sett ring rundt tallet)
0 = ingen tungpust
10 = så tungpusten det går an å bli

0 1 2 3 4 | 5 6 7 8 9 10

Se bakside

DAG 11

Dato: ....

Kl 08:00	Kl 12:00
	Husk fysioterapi-programmet før du går videre

Kl 08:00

Oksygenmetning (sett ring rundt tallet)

100 99 98 97 96 95 94 93 92 ...

Se bakside

Tungpust (sett ring rundt tallet)
0 = ingen tungpust
10 = så tungpusten det går an å bli

0 1 2 3 4 5 6 7 8 9 10

Se bakside

Pustekraft (skriv ned tallet)

....

Hvis du har hatt lav metning eller alvorlig tungpust og gjort fysioterapi, fyll ut på nytt under

Oksygenmetning (sett ring rundt tallet)

100 99 98 97 96 95 94 93 92 ...

Se bakside

Tungpust (sett ring rundt tallet)
0 = ingen tungpust
10 = så tungpusten det går an å bli

0 1 2 3 4 5 6 7 8 9 10

Se bakside

Kl 12:00

Husk fysioterapi-programmet før du går videre

Oksygenmetning (sett ring rundt tallet)

100 99 98 97 96 95 94 93 92 ...

Se bakside

Tungpust (sett ring rundt tallet)
0 = ingen tungpust
10 = så tungpusten det går an å bli

0 1 2 3 4 5 6 7 8 9 10

Se bakside

Pustekraft (skriv ned tallet)

....

Fikk du utført kl. 12:00 fysioterapi-programmet?

☐ Ja
Antall minutter: ....
☐ Nei

Hvis du har hatt lav metning eller alvorlig tungpust og gjort fysioterapi, fyll ut på nytt under

Oksygenmetning (sett ring rundt tallet)

100 99 98 97 96 95 94 93 92 ...

Se bakside

Tungpust (sett ring rundt tallet)
0 = ingen tungpust
10 = så tungpusten det går an å bli

0 1 2 3 4 5 6 7 8 9 10

Se bakside

Kl 16:00

Oksygenmetning (sett ring rundt tallet)

100 99 98 97 96 95 94 93 | 92 …

Se bakside

Tungpust (sett ring rundt tallet)
0 = ingen tungpust
10 = så tungpusten det går an å bli

0 1 2 3 4 | 5 6 7 8 9 10

Se bakside

Pustekraft (skriv ned tallet)

....

Forkjølelsessymptomer (kryss for ja)

- ☐ Feber
  Temp, hvis målt: ....
- ☐ Muskelverk
- ☐ Slapphet
- ☐ Hoste
- ☐ Sår hals
- ☐ Tett nese
- ☐ Kvalme / oppkast
- ☐ Diaré
- ☐ Redusert luktesans

Hvis du har hatt lav metning eller alvorlig tungpust og gjort fysioterapi, fyll ut på nytt under

Oksygenmetning (sett ring rundt tallet)

100 99 98 97 96 95 94 93 | 92 …

Se bakside

Tungpust (sett ring rundt tallet)
0 = ingen tungpust
10 = så tungpusten det går an å bli

0 1 2 3 4 | 5 6 7 8 9 10

Se bakside

Kl 20:00

Husk fysioterapi-programmet før du går videre

Oksygenmetning (sett ring rundt tallet)

100 99 98 97 96 95 94 93 | 92 …

Se bakside

Tungpust (sett ring rundt tallet)
0 = ingen tungpust
10 = så tungpusten det går an å bli

0 1 2 3 4 | 5 6 7 8 9 10

Se bakside

Pustekraft (skriv ned tallet)

....

Aktivitetsnivå i løpet av dagen

☐ Normalt ☐ Litt redusert ☐ Veldig redusert

Fikk du utført kl. 20:00 fysioterapi-programmet?

- ☐ Ja
  Antall minutter: ....
- ☐ Nei

Hvis du har hatt lav metning eller alvorlig tungpust og gjort fysioterapi, fyll ut på nytt under

Oksygenmetning (sett ring rundt tallet)

100 99 98 97 96 95 94 93 | 92 …

Se bakside

Tungpust (sett ring rundt tallet)
0 = ingen tungpust
10 = så tungpusten det går an å bli

0 1 2 3 4 | 5 6 7 8 9 10

Se bakside

DAG 12

Dato: ....

Kl 08:00

Oksygenmetning (sett ring rundt tallet)

100 99 98 97 96 95 94 93 | 92 ...

Se bakside

Tungpust (sett ring rundt tallet)
0 = ingen tungpust
10 = så tungpusten det går an å bli

0 1 2 3 4 | 5 6 7 8 9 10

Se bakside

Pustekraft (skriv ned tallet)

....

Hvis du har hatt lav metning eller alvorlig tungpust og gjort fysioterapi, fyll ut på nytt under

Oksygenmetning (sett ring rundt tallet)

100 99 98 97 96 95 94 93 | 92 ...

Se bakside

Tungpust (sett ring rundt tallet)
0 = ingen tungpust
10 = så tungpusten det går an å bli

0 1 2 3 4 | 5 6 7 8 9 10

Se bakside

Kl 12:00

Husk fysioterapi-programmet før du går videre

Oksygenmetning (sett ring rundt tallet)

100 99 98 97 96 95 94 93 | 92 ...

Se bakside

Tungpust (sett ring rundt tallet)
0 = ingen tungpust
10 = så tungpusten det går an å bli

0 1 2 3 4 | 5 6 7 8 9 10

Se bakside

Pustekraft (skriv ned tallet)

....

Fikk du utført kl. 12:00 fysioterapi-programmet?

☐ Ja
Antall minutter: ....
☐ Nei

Hvis du har hatt lav metning eller alvorlig tungpust og gjort fysioterapi, fyll ut på nytt under

Oksygenmetning (sett ring rundt tallet)

100 99 98 97 96 95 94 93 | 92 ...

Se bakside

Tungpust (sett ring rundt tallet)
0 = ingen tungpust
10 = så tungpusten det går an å bli

0 1 2 3 4 | 5 6 7 8 9 10

Se bakside

Kl 16:00

Oksygenmetning (sett ring rundt tallet)

100 99 98 97 96 95 94 93 | 92 ...

Se bakside

Tungpust (sett ring rundt tallet)
0 = ingen tungpust
10 = så tungpusten det går an å bli

0 1 2 3 4 | 5 6 7 8 9 10

Se bakside

Pustekraft (skriv ned tallet)

....

Forkjølelsessymptomer (kryss for ja)

- ☐ Feber
  Temp, hvis målt: ....
- ☐ Muskelverk
- ☐ Slapphet
- ☐ Hoste
- ☐ Sår hals
- ☐ Tett nese
- ☐ Kvalme / oppkast
- ☐ Diaré
- ☐ Redusert luktesans

Hvis du har hatt lav metning eller alvorlig tungpust og gjort fysioterapi, fyll ut på nytt under

Oksygenmetning (sett ring rundt tallet)

100 99 98 97 96 95 94 93 | 92 ...

Se bakside

Tungpust (sett ring rundt tallet)
0 = ingen tungpust
10 = så tungpusten det går an å bli

0 1 2 3 4 | 5 6 7 8 9 10

Se bakside

Kl 20:00

Husk fysioterapi-programmet før du går videre

Oksygenmetning (sett ring rundt tallet)

100 99 98 97 96 95 94 93 | 92 ...

Se bakside

Tungpust (sett ring rundt tallet)
0 = ingen tungpust
10 = så tungpusten det går an å bli

0 1 2 3 4 | 5 6 7 8 9 10

Se bakside

Pustekraft (skriv ned tallet)

....

Aktivitetsnivå i løpet av dagen

☐ Normalt ☐ Litt redusert ☐ Veldig redusert

Fikk du utført kl. 20:00 fysioterapi-programmet?

- ☐ Ja
  Antall minutter: ....
- ☐ Nei

Hvis du har hatt lav metning eller alvorlig tungpust og gjort fysioterapi, fyll ut på nytt under

Oksygenmetning (sett ring rundt tallet)

100 99 98 97 96 95 94 93 | 92 ...

Se bakside

Tungpust (sett ring rundt tallet)
0 = ingen tungpust
10 = så tungpusten det går an å bli

0 1 2 3 4 | 5 6 7 8 9 10

Se bakside

DAG 13

Dato: ....

Kl 08:00

Oksygenmetning (sett ring rundt tallet)

100 99 98 97 96 95 94 93 | 92 ...

Se bakside

Tungpust (sett ring rundt tallet)
0 = ingen tungpust
10 = så tungpusten det går an å bli

0 1 2 3 4 | 5 6 7 8 9 10

Se bakside

Pustekraft (skriv ned tallet)

....

Hvis du har hatt lav metning eller alvorlig tungpust og gjort fysioterapi, fyll ut på nytt under

Oksygenmetning (sett ring rundt tallet)

100 99 98 97 96 95 94 93 | 92 ...

Se bakside

Tungpust (sett ring rundt tallet)
0 = ingen tungpust
10 = så tungpusten det går an å bli

0 1 2 3 4 | 5 6 7 8 9 10

Se bakside

Kl 12:00

Husk fysioterapi-programmet før du går videre

Oksygenmetning (sett ring rundt tallet)

100 99 98 97 96 95 94 93 | 92 ...

Se bakside

Tungpust (sett ring rundt tallet)
0 = ingen tungpust
10 = så tungpusten det går an å bli

0 1 2 3 4 | 5 6 7 8 9 10

Se bakside

Pustekraft (skriv ned tallet)

....

Fikk du utført kl. 12:00 fysioterapi-programmet?

☐ Ja
Antall minutter: ....
☐ Nei

Hvis du har hatt lav metning eller alvorlig tungpust og gjort fysioterapi, fyll ut på nytt under

Oksygenmetning (sett ring rundt tallet)

100 99 98 97 96 95 94 93 | 92 ...

Se bakside

Tungpust (sett ring rundt tallet)
0 = ingen tungpust
10 = så tungpusten det går an å bli

0 1 2 3 4 | 5 6 7 8 9 10

Se bakside

Kl 16:00

Oksygenmetning (sett ring rundt tallet)

100 99 98 97 96 95 94 93 | 92 …

Se bakside

Tungpust (sett ring rundt tallet)
0 = ingen tungpust
10 = så tungpusten det går an å bli

0 1 2 3 4 | 5 6 7 8 9 10

Se bakside

Pustekraft (skriv ned tallet)

....

Forkjølelsessymptomer (kryss for ja)

- ☐ Feber
  Temp, hvis målt: ....
- ☐ Muskelverk
- ☐ Slapphet
- ☐ Hoste
- ☐ Sår hals
- ☐ Tett nese
- ☐ Kvalme / oppkast
- ☐ Diaré
- ☐ Redusert luktesans

Hvis du har hatt lav metning eller alvorlig tungpust og gjort fysioterapi, fyll ut på nytt under

Oksygenmetning (sett ring rundt tallet)

100 99 98 97 96 95 94 93 | 92 …

Se bakside

Tungpust (sett ring rundt tallet)
0 = ingen tungpust
10 = så tungpusten det går an å bli

0 1 2 3 4 | 5 6 7 8 9 10

Se bakside

Kl 20:00

Husk fysioterapi-programmet før du går videre

Oksygenmetning (sett ring rundt tallet)

100 99 98 97 96 95 94 93 | 92 …

Se bakside

Tungpust (sett ring rundt tallet)
0 = ingen tungpust
10 = så tungpusten det går an å bli

0 1 2 3 4 | 5 6 7 8 9 10

Se bakside

Pustekraft (skriv ned tallet)

....

Aktivitetsnivå i løpet av dagen

☐ Normalt ☐ Litt redusert ☐ Veldig redusert

Fikk du utført kl. 20:00 fysioterapi-programmet?

- ☐ Ja
  Antall minutter: ....
- ☐ Nei

Hvis du har hatt lav metning eller alvorlig tungpust og gjort fysioterapi, fyll ut på nytt under

Oksygenmetning (sett ring rundt tallet)

100 99 98 97 96 95 94 93 | 92 …

Se bakside

Tungpust (sett ring rundt tallet)
0 = ingen tungpust
10 = så tungpusten det går an å bli

0 1 2 3 4 | 5 6 7 8 9 10

Se bakside

DAG 14

Dato: ....

Kl 08:00

Oksygenmetning (sett ring rundt tallet)

100 99 98 97 96 95 94 93 | 92 ...

Se bakside

Tungpust (sett ring rundt tallet)

0 = ingen tungpust
10 = så tungpusten det går an å bli

0 1 2 3 4 | 5 6 7 8 9 10

Se bakside

Pustekraft (skriv ned tallet)

....

Hvis du har hatt lav metning eller alvorlig tungpust og gjort fysioterapi, fyll ut på nytt under

Oksygenmetning (sett ring rundt tallet)

100 99 98 97 96 95 94 93 | 92 ...

Se bakside

Tungpust (sett ring rundt tallet)

0 = ingen tungpust
10 = så tungpusten det går an å bli

0 1 2 3 4 | 5 6 7 8 9 10

Se bakside

Kl 12:00

Husk fysioterapi-programmet før du går videre

Oksygenmetning (sett ring rundt tallet)

100 99 98 97 96 95 94 93 | 92 ...

Se bakside

Tungpust (sett ring rundt tallet)

0 = ingen tungpust
10 = så tungpusten det går an å bli

0 1 2 3 4 | 5 6 7 8 9 10

Se bakside

Pustekraft (skriv ned tallet)

....

Fikk du utført kl. 12:00 fysioterapi-programmet?

☐ Ja

Antall minutter: ....

☐ Nei

Hvis du har hatt lav metning eller alvorlig tungpust og gjort fysioterapi, fyll ut på nytt under

Oksygenmetning (sett ring rundt tallet)

100 99 98 97 96 95 94 93 | 92 ...

Se bakside

Tungpust (sett ring rundt tallet)

0 = ingen tungpust
10 = så tungpusten det går an å bli

0 1 2 3 4 | 5 6 7 8 9 10

Se bakside

Kl 16:00

Oksygenmetning (sett ring rundt tallet)

100 99 98 97 96 95 94 93 | 92 ...

Se bakside

Tungpust (sett ring rundt tallet)
0 = ingen tungpust
10 = så tungpusten det går an å bli

0 1 2 3 4 | 5 6 7 8 9 10

Se bakside

Pustekraft (skriv ned tallet)

....

Forkjølelsessymptomer (kryss for ja)

- ☐ Feber
  Temp, hvis målt: ....
- ☐ Muskelverk
- ☐ Slapphet
- ☐ Hoste
- ☐ Sår hals
- ☐ Tett nese
- ☐ Kvalme / oppkast
- ☐ Diaré
- ☐ Redusert luktesans

Hvis du har hatt lav metning eller alvorlig tungpust og gjort fysioterapi, fyll ut på nytt under

Oksygenmetning (sett ring rundt tallet)

100 99 98 97 96 95 94 93 | 92 ...

Se bakside

Tungpust (sett ring rundt tallet)
0 = ingen tungpust
10 = så tungpusten det går an å bli

0 1 2 3 4 | 5 6 7 8 9 10

Se bakside

Kl 20:00

Husk fysioterapi-programmet før du går videre

Oksygenmetning (sett ring rundt tallet)

100 99 98 97 96 95 94 93 | 92 ...

Se bakside

Tungpust (sett ring rundt tallet)
0 = ingen tungpust
10 = så tungpusten det går an å bli

0 1 2 3 4 | 5 6 7 8 9 10

Se bakside

Pustekraft (skriv ned tallet)

....

Aktivitetsnivå i løpet av dagen

☐ Normalt ☐ Litt redusert ☐ Veldig redusert

Fikk du utført kl. 20:00 fysioterapi-programmet?

- ☐ Ja
  Antall minutter: ....
- ☐ Nei

Hvis du har hatt lav metning eller alvorlig tungpust og gjort fysioterapi, fyll ut på nytt under

Oksygenmetning (sett ring rundt tallet)

100 99 98 97 96 95 94 93 | 92 ...

Se bakside

Tungpust (sett ring rundt tallet)
0 = ingen tungpust
10 = så tungpusten det går an å bli

0 1 2 3 4 | 5 6 7 8 9 10

Se bakside

FLYTSKJEMA FOR MÅLING AV METNING

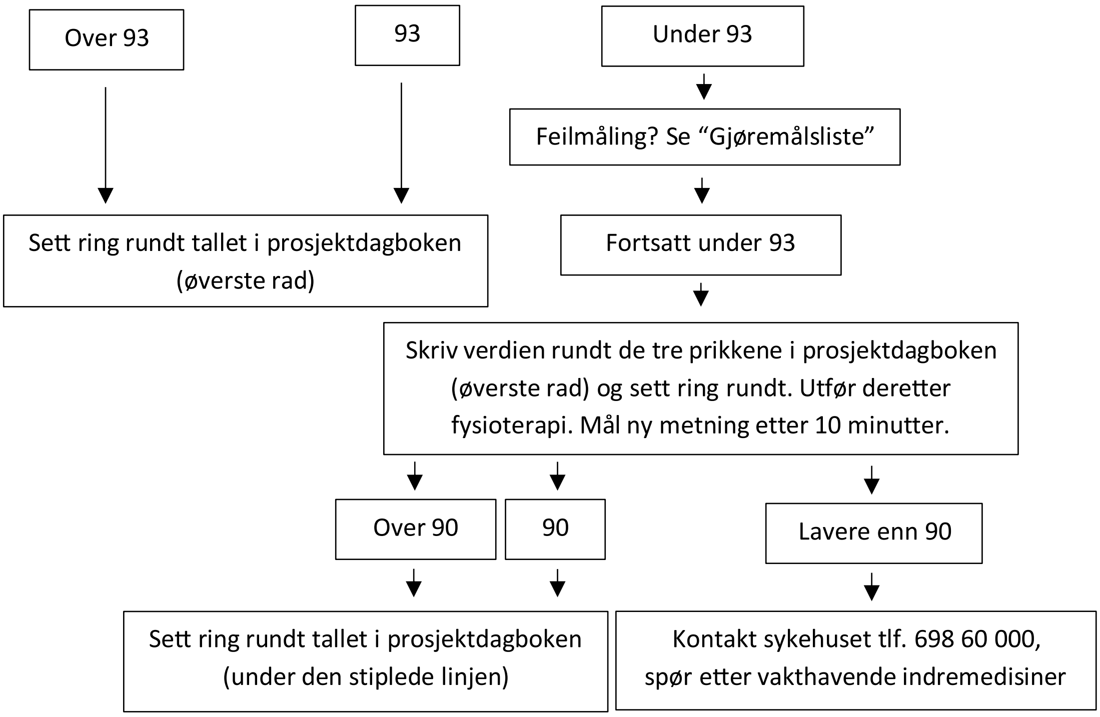

GJØREMÅLSLISTE VED MISTANKE OM FEILMÅLING AV METNING

1) Fjerne neglelakk
2) Varme hånden/fingrene
3) Ta av metningsmåleren og plasser det på en annen finger på den andre hånden, pass på at armen er under hjertehøyde
4) Skru opp belysningen eller gå inn i et annet rom med bedre belysning

FLYTSKJEMA FOR MÅLING AV TUNGPUST

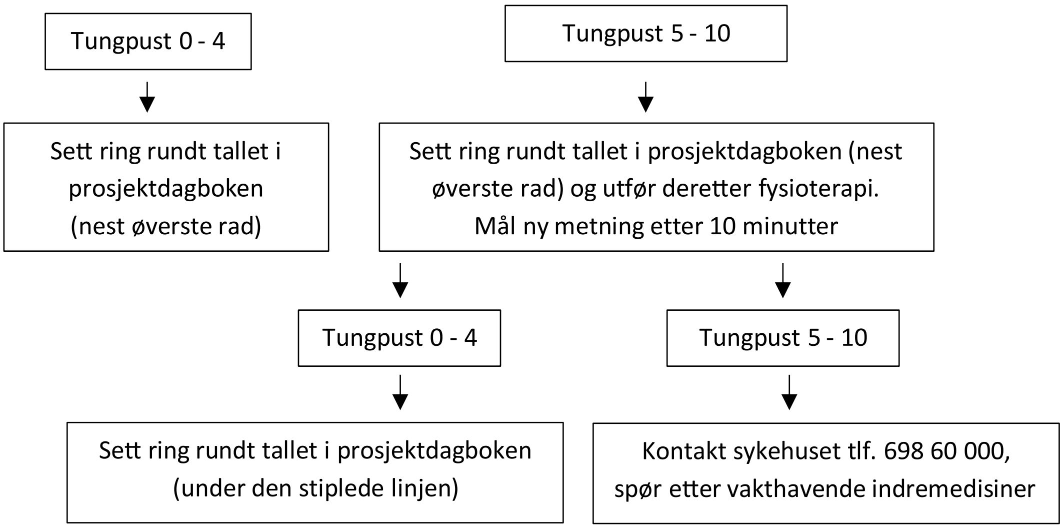

Supplement: Supplementary file 4 — Additional file 4. [file 40814_2023_1415_MOESM4_ESM.pdf]
